# Synthesis of Polysubstituted Tetrahydropyrans by Stereoselective Hydroalkoxylation of Silyl Alkenols: En Route to Tetrahydropyranyl Marine Analogues

**DOI:** 10.3390/md16110421

**Published:** 2018-11-01

**Authors:** Carlos Díez-Poza, Patricia Val, Francisco J. Pulido, Asunción Barbero

**Affiliations:** Department of Organic Chemistry, Faculty of Science, Campus Miguel Delibes, 47011 Valladolid, Spain; solracdp@hotmail.com (C.D.-P.); patrivaldo@hotmail.com (P.V.); pulido@qo.uva.es (F.J.P.)

**Keywords:** tetrahydropyrans, acid mediated cyclization, stereoselective, marine drugs analogues

## Abstract

Tetrahydropyrans are abundantly found in marine natural products. The interesting biological properties of these compounds and their analogues make necessary the development of convenient procedures for their synthesis. In this paper, an atom economy access to tetrahydropyrans by intramolecular acid-mediated cyclization of silylated alkenols is described. p-TsOH has shown to be an efficient reagent to yield highly substituted tetrahydropyrans. Moreover, excellent diastereoselectivities are obtained both for unsubstituted and alkylsubstituted vinylsilyl alcohols. The methodology herein developed may potentially be applied to the synthesis of marine drugs derivatives.

## 1. Introduction

**Figure 1:**
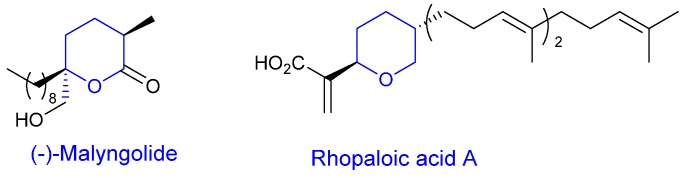
Illustrative examples of THP-containing marine natural products.

Tetrahydropyrans are interesting building blocks present in a great number of bioactive marine natural products. Some representative examples include 2,2,5-trisubstituted tetrahydropyrans such as malyngolide [1], antibiotic collected from the lipid extract of cyanobacteria *Lyngbya majuscula* which shows activity against *M. Smegmatis* and *Streptococcus pyogenes*, or 2,5-disubstituted tetrahydropyrans such as rhopaloic acids A and B, which have been isolated from the marine sponge *Rhopaloeides* sp. and show potent inhibitory activity against the embryonic development of the starfish *Asterina pectinifera* [2] (Figure 1). The interesting biological properties of these scaffolds together with their scarce availability has prompted scientists to develop approaches for their synthesis. Moreover, total synthesis has the advantage of enabling the introduction of structural alterations in the molecule for the preparation of analogues with potential biological properties.

**Scheme 1:**
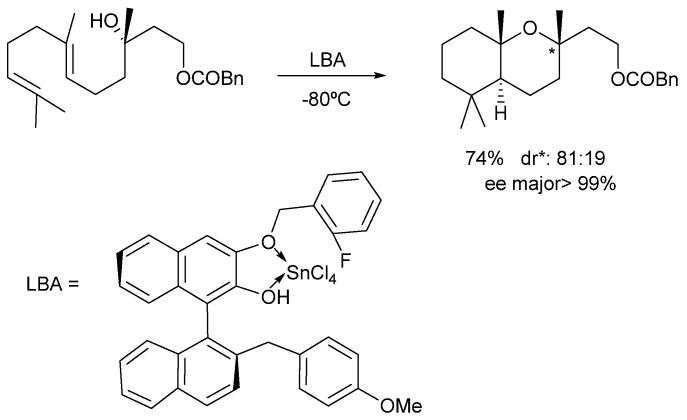
Towards the synthesis of natural caparrapi oxide.

The synthesis of these marine products and analogues requires an effective procedure for the stereoselective construction of the tetrahydropyran ring. Different strategies have been devised for the formation of these six membered rings including nucleophilic substitution ring formation, epoxide-mediated annulations or alkene-mediated cyclizations [3,4]. Within the stoichiometric alkene-mediated cyclizations the most common electrophiles used to activate the alkene addition are mercury salts, halogens and seleno reagents. However, few number of approaches have been described on the intramolecular trapping of an alkene by an oxygenated nucleophile in the presence of an appropriate Brønsted [5,6,7] or Lewis acid [8,9,10]. Although this protocol could be considered a very efficient atom economy process, the strategy has generally proven to be of limited utility due to frequent side reactions and lack of generality [11,12]. Moreover, as far as we know, most of the described methods are focused on the regioselective formation of tetrahydropyrans with no more than one stereogenic centre. However, very few examples examine the stereoselective aspects of these processes, usually representing specific key transformations in a total synthesis rather than a general established methodology. For instance, Yamamoto has used this protocol in the synthesis of natural caparrapi oxide, where the key step is the stoichiometric acid catalysed cyclization of the corresponding γ-hydroxy alkene [13] (Scheme 1).

**Scheme 2:**
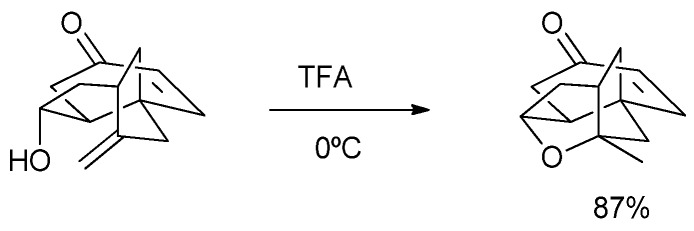
Towards the synthesis of natural platensimycin.

In another example Nicolaou [14] has reported the formation of the cage-like structure of natural platensimycin by the acid catalysed cyclization of the corresponding alkenol (Scheme 2).

Therefore, there is a need to develop a general and efficient method for the stereoselective synthesis of tetrahydropyrans through the acid-mediated cyclization of alkenols.

For many years our research group has been devoted to exploring new synthetic approaches to the synthesis of carbo- [15,16,17] and heterocycles [18,19] using the chemistry of organosilanes. Lately, Hosomi [20,21] and our group [22,23] have reported an efficient synthesis of tetrahydrofurans by intramolecular hydroalkoxylation reaction of vinyl or allylsilanes. In this paper, we report the extension of this work to the stereoselective synthesis of polysubstituted tetrahydropyrans by the acid-mediated cyclization of activated vinylsilyl alcohols.

## 2. Results

**Scheme 3:**
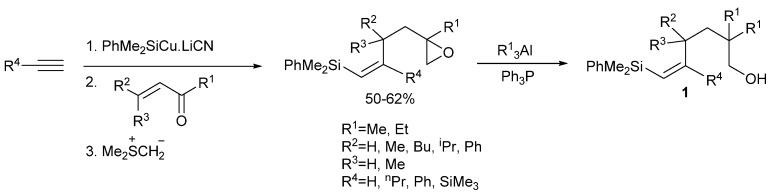
Preparation of the starting vinylsilyl alcohols.

The δ-hydroxy alkenes needed for this study were readily prepared in three steps such as: silylcupration of alkynes and reaction with α,β-unsaturated ketones, formation of the epoxide derivative using sulphur ylides and final S_N_1 opening of the epoxide with trialkylaluminum reagents [24] to obtain the corresponding primary alcohol. Unfortunately, primary alcohols **1** showed to be unstable under chromatography conditions, which made necessary to perform the following cyclization without previous purification (Scheme 3).

We used vinylsilyl alcohol **1a** as a model study to get the optimized conditions for this cyclization, using various Lewis and Brønsted acids. The results are shown in Table 1.

p-TsOH showed to be the most efficient reagent for this cyclization (Table 1, entry 10). The reaction in the presence of TMSOTf or TiCl_4_ gave a complex mixture (Table 1, entries 1–3), while no reaction was observed using ZnCl_2_ or under a suspension of silica (Table 1, entries 4 and 5). BF_3_·OEt_2_ was not effective to promote the cyclization at −78 °C, although a 2:1 mixture of both possible stereoisomers was obtained in moderate yield when the reaction temperature was increased to 0 °C (Table 1, entries 6 and 7). Similarly, SnCl_4_ mediated cyclization provided at −78 °C a 1:1 mixture of isomers in reasonable yield (Table 1, entry 8). Regarding the use of Brønsted acids, the reaction in the presence of CSA resulted to be extremely slow, while the best results were obtained by refluxing the alcohol in the presence of p-TsOH for 1 hour (Table 1, entry 10). Under these conditions tetrahydropyran **2a** was obtained in high yield and excellent diastereoselectivity (a single isomer was observed in the reaction mixture). 

With these results in hand and in order to broaden the structural diversity of the tetraydropyrans obtained by this methodology, we decided to apply the optimized conditions (Table 1, entry 10) to different vinylsilyl alcohols. The results are summarized in Table 2.

As shown, the process is high yielding and very stereoselective (both for unsubstituted and alkylsubstituted vinylsilyl alcohols) under p-TsOH mediation at reflux temperature. The short reaction time needed to reach completion may indicate the benefit of the gem-dialkyl group (Thorpe-Ingold effect), since the only example of preparation of a THP (a non-substituted one) reported by Hosomi required reflux for 96 hours [21]. The use of stoichiometric amounts of p-TsOH was proven to be the best choice, since under lower loadings of acid the reaction is slower and a certain amount of the starting vinylsilyl alcohol is always recovered (Table 2, entry 2). Moreover, at room temperature the process requires prolonged reaction time which results in lower yields due to partial protodesilylation [25] of the cyclization products (Table 2, entry 3). Furthermore, the reaction seems to be dependent on steric effects, since reaction of alcohol **1h** bearing a vinylic bulky group, such as Me_3_Si, did not react under the shown conditions (Table 2, entry 10).

We then decided to study the effect of a phenyl group β to silicon in the rate and selectivity of the cyclization. Vinylsilyl alcohol **1i** was chosen as a model substrate for the cyclization process (Table 3).

As expected, the cyclization rate is significantly enhanced by the introduction of the phenyl group in the starting vinylsilyl alcohol. Thus, at reflux temperature the cyclization of **1i** under p-TsOH induction provides only the corresponding desilylated tetrahydropyrans (Table 3, entry 3) while at room temperature a mixture of both silylated and desilylated tetrahydropyrans are obtained. Finally, at 0 °C the reaction is fast enough to produce the cyclization products in 10 min, without side desilylative processes (Table 3, entry 5). Even CSA (either at room temperature or at 0 °C) is reactive enough to provide in good yields the cyclization tetrahydropyrans without any side product (Table 3, entries 6 and 7). 

We then studied the scope of the process employing different starting alcohols under the optimized conditions (Table 3, entry 5). The results are shown in Table 4. 

Although the cyclization of alcohols **1i**–**l** occurs in good yields, the stereoselectivity of the process is decreased, obtaining mixtures of both possible diastereoisomers, in which the stereoisomer with the silylmethyl group *anti* to the R^2^ substituent is always predominant. The best stereoselectivity is obtained when a bulky R^2^ is present at C-3 (Table 4, entry 3), which indicates that the reaction is influenced by steric effects.

A mechanism that could account for these cyclizations implies an initial protonation of the alcohol, which in turn would deliver the proton to the alkene moiety. The formation of a stabilized β to silicon carbocation will be followed by intramolecular attack of the hydroxyl group to form the tetrahydropyranyl ring.

Regarding the stereoselectivity of the process, one single diastereoisomer is obtained when R^4^ is either a hydrogen or an alkyl group. However, a certain loss of stereocontrol is observed when R^4^ is a phenyl group (Table 4, entries 1–4). In either case the unique or major isomer is the one in which the silylmethyl group is *anti* to the C-3 substituent. 

**Figure 2:**
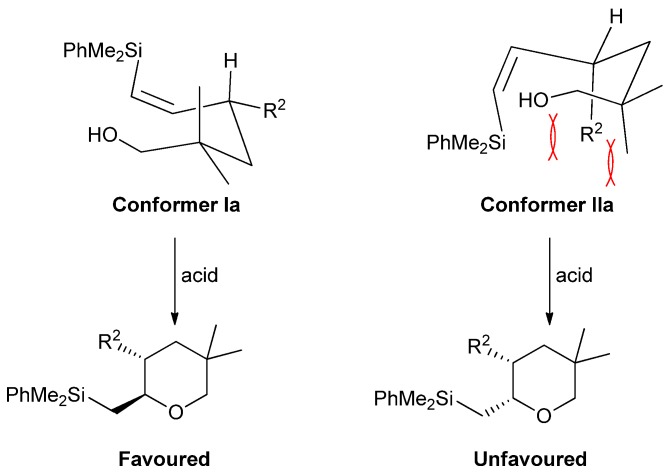
Stereochemical outcome of cyclization of alcohols **1a**–**e**.

In accordance with Fleming [26,27] and Hook’s [28] models for the reaction of electrophiles with alkenes bearing an allylic stereogenic centre, two different chair-like conformations (**Ia** and **IIa**) could be drawn for vinylsilyl alcohols **1a**–**e**. In the preferred conformation **Ia** the allylic hydrogen is partially eclipsing the double bond (“inside”), while the largest substituent is antiperiplanar to the alkene moiety (Figure 2). The alternative conformation **IIa** (with R^2^ inside) shows a disfavoured 1,3-allylic interaction between R^2^ and the silyl group and a 1,3-diaxial interaction between R^2^ and the Me group, which would explain the preferred formation of 2,3-*trans*-tetrahydropyranes **2a**–**e**.

**Figure 3:**
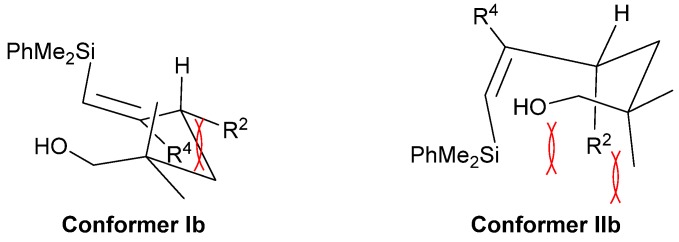
Chair-like reactive conformations for arylsubstituted vinylsilyl alcohols **1i**–**l**.

The decrease in stereoselectivity observed for arylsubstituted vinylsilyl alcohols **1i**–**l** could be explained using the same model, since now, besides a disfavoured 1,3-diaxial interaction in conformer **IIb**, there is a competing 1,2-allylic interaction between R^2^ and R^4^ in conformer **Ib**. This interaction is especially strong when the phenyl group is coplanar with the double bond, while the possibility of rotating around would cause the loss of the resonance stabilization. For alcohols **1f**–**g** R^4^ is a flexible alkyl chain and this 1,2-allylic interaction seems to be rather small (Figure 3).

**Scheme 4:**
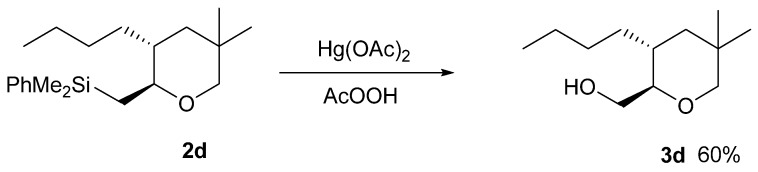
Synthesis of 2-hydroxymethyltetrahydropyran **3d**.

In addition, the presence of a remaining silyl group in the final tetrahydropyrans offers the attractive possibility of further functionalization. As known, Fleming-Tamao oxidation [29] permits the transformation of the silyl group to a hydroxy group. To demonstrate the potential of these tetrahydropyrans as key intermediates for the synthesis of tetrahydropyranyl marine natural products and their synthetic analogues, we have transformed tetrahydropyran **2d** into the corresponding alcohol **3d** [30] (Scheme 4).

**Scheme 5:**
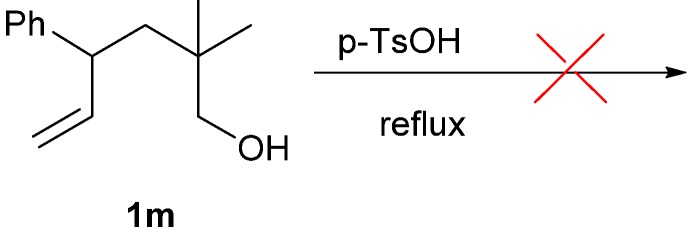
Influence of the silyl group in the cyclization.

Finally, we decided to study the effect of the silyl group in the cyclization of silyl alkenols **1**. For this purpose, we synthesized an analogue of alcohol **1e** lacking the silyl moiety (**1m**). Reaction of alcohol **1m** with p-TsOH in DCM did not occur at r.t., nor under reflux conditions, recovering after 5 hours the unreacted starting alcohol. This seems to indicate that the presence of an electron-rich alkene, such as the vinylsilane, is needed for the cyclization to proceed (Scheme 5).

## 3. Materials and Methods 

### 3.1. General Procedures for the Acid-catalysed Cyclization of Vinylsilyl Alcohols

To a solution of the acid (1 mmol) in dry CH_2_Cl_2_ (10 mL) is added a solution of the alcohol (1 mmol) in CH_2_Cl_2_. The mixture is stirred under N_2_ in the shown conditions (Table 1, Table 2, Table 3 and Table 4) and quenched with saturated solution of NaHCO_3_ (5 mL). The organic layer was washed 3 times with NaHCO_3_, dried over MgSO_4_, evaporated in vacuo and purified by flash chromatography (EtOAc/hexane). 

### 3.2. Trans-5,5-Dimethyl-2-Dimethylphenylsilylmethyl-3-Phenyl-Tetrahydropyran *(**2a**)*

Colourless oil (77%); ^1^H NMR (400 MHz, CDCl_3_) δ = 7.49–7.45 (m, 2H), 7.38–7.34 (m, 3H), 7.33–7.24 (m, 3H), 7.14–7.11 (m, 2H), 3.56 (dd, *J* = 11.4 and 2.4 Hz, 1H), 3.43 (td, *J* = 9.7 and 3.5 Hz, 1H), 3.23 (d, *J* = 11.4 Hz, 1H), 2.73–2.64 (m, 1H), 1.67–1.59 (m, 1H), 1.54 (t, *J* = 13.0 Hz, 1H), 1.19 (s, 3H), 0.92–0.87 (m, 2H, CH_2_-Si), 0.88 (s, 3H), 0.33 (s, 3H, CH_3_-Si), 0.29 (s, 3H, CH_3_-Si); ^13^C NMR (101 MHz, CDCl_3_) δ = 143.9 (C), 140.0 (C), 133.7 (CH), 128.6 (CH), 128.0 (CH), 127.6 (CH), 126.3 (CH), 80.4 (CH), 78.2 (CH_2_), 48.0 (CH), 46.3 (CH_2_), 31.0 (C), 27.2 (CH_3_), 24.2 (CH_3_), 20.8 (CH_2_-Si), −1.5 (CH_3_), −2.5 (CH_3_); HRMS (ESI+) *m/z* calcd for C_22_H_30_NaOSi ([M + Na]^+^): 361.1958, found 361.1957.

### 3.3. 5,5-Dimethyl-2-Dimethylphenylsilylmethyl-Tetrahydropyran *(**2b**)*

Colourless oil (71%); ^1^H NMR (400 MHz, CDCl_3_) δ = 7.63–7.56 (m, 2H), 7.45–7.38 (m, 3H), 3.46 (dd, *J* = 11.0 and 2.2 Hz, 1H), 3.35–3.26 (m, 1H), 3.14 (d, *J* = 11.0 Hz, 1H), 1.51–1.42 (m, 3H), 1.36–1.24 (m, 1H), 1.23–1.18 (m, 1H), 1.09 (dd, *J* = 14.5 and 7.0 Hz, 1H), 1.04 (s, 3H), 0.81 (s, 3H), 0.37 (s, 3H, CH_3_-Si), 0.35 (s, 3H, CH_3_-Si); ^13^C NMR (101 MHz, CDCl_3_) δ = 139.5 (C), 133.6 (CH), 128.8 (CH), 127.7 (CH), 78.2 (CH_2_), 75.9 (CH), 37.1 (CH_2_), 31.2 (CH_2_), 29.6 (C), 27.2 (CH_3_), 24.1 (CH_2_), 23.6 (CH_3_), −1.8 (CH_3_), −2.2 (CH_3_); HRMS (ESI+) *m/z* calcd for C_16_H_26_NaOSi ([M + Na]^+^): 285.1645, found 285.1646.

### 3.4. 3,3,5,5-Tetramethyl-2-Dimethylphenylsilylmethyl-Tetrahydropyran *(**2c**)*

Colourless oil (70%); ^1^H NMR (400 MHz, CDCl_3_) δ = 7.59–7.53 (m, 2H), 7.39–7.31 (m, 3H), 3.49 (dd, *J* = 11.1 and 2.5 Hz, 1H), 2.99–2.94 (m, 2H), 1.35 (dd, *J* = 13.6 and 2.5 Hz, 1H), 1.17–1.12 (m, 1H), 1.10 (s, 3H), 1.01 (s, 3H), 0.94–0.88 (m, 2H), 0.76 (s, 3H), 0.73 (s, 3H), 0.35 (s, 3H, CH_3_-Si), 0.33 (s, 3H, CH_3_-Si); ^13^C NMR (101 MHz, CDCl_3_) δ = 140.2 (C), 133.8 (CH), 128.6 (CH), 127.6 (CH), 84.0 (CH), 79.1 (CH_2_), 52.2 (CH_2_), 34.1 (C), 31.3 (C), 29.6 (CH_3_), 29.3 (CH_3_), 26.5 (CH_3_), 21.6 (CH_3_), 16.0 (CH_2_), −1.2 (CH_3_), −2.8 (CH_3_); HRMS (ESI+) *m/z* calcd for C_18_H_30_NaOSi ([M + Na]^+^): 313.1958, found 313.1956.

### 3.5. Trans-3-Butyl-5,5-Dimethyl-2-Dimethylphenylsilylmethyl-Tetrahydropyran *(**2d**)*

Colourless oil (73%); ^1^H NMR (400 MHz, CDCl_3_) δ = 7.66–7.60 (m, 2H), 7.45–7.39 (m, 3H), 3.45 (dd, *J* = 11.0 and 2.6 Hz, 1H), 3.02 (d, *J* = 11.0 Hz, 1H), 2.94 (td, *J* = 10.0 and 2.8 Hz, 1H), 1.61–1.56 (m, 1H) 1.50–1.20 (m,8H), 1.06 (s, 3H), 1.00–0.91 (m, 2H), 0.93 (t, *J* = 7.0, 3H) 0.83 (s, 3H), 0.38 (s, 6H, (CH_3_)_2_-Si); ^13^C NMR (101 MHz, CDCl_3_) δ = 140.5 (C), 133.7 (CH), 128.6 (CH), 127.6 (CH), 80.9 (CH), 77.8 (CH_2_), 43.2 (CH_2_), 39.4 (CH), 31.8 (CH_2_), 30.9 (C), 28.4 (CH_2_), 27.4 (CH_3_), 24.4 (CH_3_), 23.0 (CH_2_), 20.8 (CH_2_), 14.1 (CH_3_), −1.4 (CH_3_), −2.4 (CH_3_); HRMS (ESI+) *m/z* calcd for C_20_H_34_NaOSi ([M + Na]^+^): 341.2271, found 341.2271.

### 3.6. Trans-3-Isopropy-5,5-Dimethyl-2-Dimethylphenylsilylmethyl-Tetrahydropyran *(**2e**)*

Colourless oil (75%); ^1^H NMR (400 MHz, CDCl_3_) δ = 7.55–7.52 (m, 2H), 7.34–7.32 (m, 3H), 3.35 (dd, *J* = 11.0 and 2.7 Hz, 1H), 3.06 (td, *J* = 10.3 and 3.1 Hz, 1H), 2.92 (d, *J* = 11.0, 1H), 1.89–1.82 (m, 1H), 1.38–1.32 (m, 1H), 1.30–1.25 (m, 1H), 1.17 (dd, *J* = 14.8 and 3.1 Hz, 1H), 0.98 (s, 3H, CH_3_), 1.00–0.97 (m, 1H), 0.90 (dd, *J* = 14.8 and 10.3 Hz, 1H), 0.81 (d, *J* = 7.0 Hz, 3H, CH_3_), 0.78 (s, 3H, CH_3_), 0.63 (d, *J* = 6.9 Hz, 3H, CH_3_), 0.31 (s, 3H, CH_3_-Si), 0.30 (s, 3H, CH_3_-Si);^13^C NMR (101 MHz, CDCl_3_) δ = 140.5 (C), 133.6 (CH), 128.5 (CH), 127.6 (CH), 79.1 (CH), 77.9 (CH_2_), 44.1 (CH), 35.7 (CH_2_), 30.7 (C), 27.6 (CH), 26.9 (CH_3_), 24.4 (CH_3_), 21.0 (CH_3_), 20.3 (CH_2_-Si), 15.3 (CH_3_), −1.4 (CH_3_), −2.5 (CH_3_); HRMS (ESI+) *m/z* calcd for C_19_H_32_NaOSi ([M + Na]^+^): 327.2110, found 327.2115.

### 3.7. 5,5-Dimethyl-2-Dimethylphenylsilylmethyl-3-Phenyl-2-Propyl-Tetrahydropyran *(**2f**)*

Colourless oil (71%); ^1^H NMR (400 MHz, CDCl_3_) δ = 7.58–7.19 (m, 10H), 3.21 (d, *J* = 11.6, 1H), 3.15 (dd, *J* = 11.6 and 2.4 Hz, 1H), 3.00 (dd, *J* = 13.5 and 3.7 Hz, 1H), 2.17–2.06 (m, 1H), 2.01 (t, *J* = 13.5 Hz, 1H), 1.24–1.17 (m, 2H), 1.34 (dt, *J* = 13.5 and 3.5 Hz,1H), 1.23 (d, *J* = 15.4, 1H, CH*H*Si), 1.13 (d, *J* = 15.4, 1H, CH*H*Si), 1.00 (s, 3H), 0.97–0.92 (m, 1H),0.88 (d, *J* = 7.3 Hz, 3H), 0.85 (s, 3H), 0.50 (s, 3H, CH_3_-Si), 0.35 (s, 3H, CH_3_-Si); ^13^C NMR (101 MHz, CDCl_3_) δ = 142.9 (C), 141.8 (C), 133.7 (CH), 129.7 (CH), 128.3 (CH), 127.8 (CH), 127.5 (CH), 126.3 (CH), 78.8 (C), 71.1 (CH_2_), 48.4 (CH), 39.9 (CH_2_), 32.8 (CH_2_), 30.8 (C), 27.8 (CH_3_), 26.6 (CH_2_-Si), 24.8 (CH_3_), 15.4 (CH_2_), 14.7 (CH_3_), −0.3 (CH_3_), −0.4 (CH_3_); HRMS (ESI+) *m/z* calcd for C_25_H_36_NaOSi ([M + Na]^+^): 406.2428, found 403.2435.

### 3.8. 5,5-Diethyl-3-Methyl-2-Dimethylphenylsilylmethyl-2-Propyl-Tetrahydropyran *(**2g**)*

Colourless oil (70%); ^1^H NMR (400 MHz, CDCl_3_) δ = 7.54–7.31 (m, 5H), 3.17 (d, *J* = 11.7 Hz, 1H), 3.08 (dd, *J* = 11.7 Hz, 1H), 1.95–1.89 (m, 1H), 1.55–1.41 (m, 7H), 1.31–1.24 (m, 3H), 1.07–0.99 (m, 2H), 0.90–0.82 (m 6H), 0.80 (t, *J* = 7.0 Hz, 3H), 0.73 (t, *J* = 7.5 Hz, 3H), 0.67 (d, *J* = 6.9 Hz, 3H), 0.37 (s, 3H, CH_3_-Si), 0.31 (s, 3H, CH_3_-Si); ^13^C NMR (101 MHz, CDCl_3_) δ = 141.2 (C), 133.4 (CH), 128.5 (CH), 127.6 (CH), 79.8 (C), 69.0 (CH_2_), 43.2 (CH_2_), 37.1 (CH_2_), 35.5 (C), 30.4 (CH), 28.7 (CH_2_), 23.6 (CH_2_), 17.6 (CH_3_), 17.1 (CH_2_), 16.1 (CH_2_), 14.6 (CH_3_), 7.5 (CH_3_), 6.9 (CH_3_), −0.4 (CH_3_), −1.0 (CH_3_); HRMS (ESI+) *m/z* calcd for C_22_H_38_NaOSi ([M + Na]^+^): 369.2584, found 369.2582.

### 3.9. 3,5,5-Trimethyl-2-Dimethylphenylsilylmethyl-2-Phenyl-Tetrahydropyrans ***2i*** and ***3i***

Chromatography gave tetrahydrofurans **2i** and **3i** as a mixture. Colourless oil (71%).

(**2i**)*:* ^1^H NMR (400 MHz, CDCl_3_) δ = 7.53–7.51 (m, 2H), 7.49–7.46 (m, 2H), 7.34–7.26 (m, 5H), 7.23–7.19 (m, 1H), 3.26 (d, *J* = 11.8, 1H), 3.19 (dd, *J* = 11.8 and 2.3 Hz, 1H), 1.79–1.73 (m, 1H), 1.74 (d, *J* = 15.4 Hz, 1H, C*H*HSi), 1.44 (d, *J* = 15.4 Hz, 1H, CH*H*Si), 1.35–1.23 (m, 2H), 1.13 (s, 3H), 0.80 (s, 3H), 0.68 (d, *J* = 6.8 Hz, 3H), 0.14 (s, 3H, CH_3_-Si), −0.13 (s, 3H, CH_3_-Si); ^13^C NMR (101 MHz, CDCl_3_) δ = 147.5 (C), 141.1 (C), 133.4 (CH), 128.5 (CH), 127.6 (CH), 127.4 (CH), 126.3 (CH), 126.1 (CH), 81.6 (C), 71.9 (CH_2_), 42.7 (CH_2_), 39.0 (CH), 30.9 (C), 27.4 (CH_3_), 24.2 (CH_3_), 17.2 (CH_3_), 13.7 (CH_2_-Si), −1.4 (CH_3_), −1.5 (CH_3_); HRMS (ESI+) *m/z* calcd for C_23_H_32_OSi ([M + Na]^+^): 375.2110, found 375.2115.

(**3i**): distinguishable signals: ^1^H NMR (400 MHz, CDCl_3_) δ = 3.32 (d, *J* = 11.7, 1H), 2.07–2.00 (m, 1H), 0.96 (s, 3H), 0.89 (s, 3H), 0.78 (d, *J* = 7.2 Hz, 3H), 0.22 (s, 3H), 0.07 (s, 3H); ^13^C NMR (101 MHz, CDCl_3_) δ = 28.2 (CH_3_), 26.4 (CH_3_), 19.0 (CH_3_).

### 3.10. 5,5-Dimethyl-2-Dimethylphenylsilylmethyl-2,3-Diphenyl-Tetrahydropyrans ***2j*** and ***3j***

Chromatography gave tetrahydrofurans **2j** and **3j** as a mixture. Colourless oil (65%).

(**2j**): ^1^H NMR (400 MHz, CDCl_3_) δ = 7.44–7.13 (m, 13H), 6.74–6.71 (m, 2H), 3.46 (d, *J* = 11.8, 1H), 3.31 (dd, *J* = 11.8 and 2.3 Hz, 1H), 2.98 (dd, *J* = 13.7 and 1.5 Hz, 1H), 2.12–2.05 (m, 2H, CH*H*Si), 1.49 (dt, *J* = 13.6 and 2.8 Hz, 1H), 1.27 (d, *J* = 14.9 Hz, 1H, C*H*HSi), 1.22 (s, 3H), 0.92 (s, 3H), 0.13 (s, 3H, CH_3_-Si), −0.20 (s, 3H, CH_3_-Si); ^13^C NMR (101 MHz, CDCl_3_) δ = 146.29 (C), 141.56 (C), 140.9 (C), 133.4 (CH), 129.9 (CH), 128.5 (CH), 127.6 (CH), 127.2 (CH), 126.9 (CH), 126.4 (CH), 126.4 (CH), 126.3 (CH), 81.4 (C), 71.7 (CH_2_), 51.9 (CH), 40.3 (CH_2_), 31.1 (C), 27.5 (CH_3_), 24.0 (CH_3_), 15.4 (CH_2_-Si), −1.4 (CH_3_), −1.5 (CH_3_); HRMS (ESI+) *m/z* calcd for C_28_H_35_OSi ([M + H]^+^): 415.2450, found 415.2452.

(**3j**): distinguishable signals: ^1^H NMR (400 MHz, CDCl_3_) δ = 3.52 (d, *J* = 11.9, 1H), 3.38 (dd, *J* = 11.9 and 1.5 Hz, 1H), 3.16 (dd, *J* = 13.6 and 2.1 Hz, 1H), 1.89 (d, *J* = 14.9 Hz, 1H, C*H*HSi), 1.84 (d, *J* = 14.9 Hz, 1H, CH*H*Si), 1.75 (dd, *J* = 13.6 and 13.1 Hz, 1H), 1.22 (s, 3H), 0.96 (s, 3H), 0.14 (s, 3H, CH_3_-Si), −0.26 (s, 3H, CH_3_-Si); ^13^C NMR (101 MHz, CDCl_3_) δ = 129.3 (CH), 128.3 (CH), 127.9 (CH), 127.5 (CH), 127.23 (CH), 126.5 (CH), 126.2 (CH), 126.1 (CH), 82.9 (C), 70.6 (CH_2_), 51.8 (CH), 38.2 (CH_2_), 32.8 (C), 29.3 (CH_3_), 28.1 (CH_2_), 25.0 (CH_3_), −2.1 (CH_3_).

### 3.11. 3-Isopropyl-5,5-Dimethyl-2-Dimethylphenylsilylmethyl-2-Phenyl-Tetrahydropyrans ***2k*** and ***3k***

Chromatography gave tetrahydrofurans **2k** and **3k** as a mixture. Colourless oil (72%).

(**2k**)*:*^1^H NMR (400 MHz, CDCl_3_) δ = 7.52–7.19 (m, 10H), 3.23 (d, *J* = 11.6, 1H), 3.15 (dd, *J* = 11.6 and 2.3 Hz, 1H), 1.85 (d, *J* = 15.3, 1H, C*H*HSi), 1.60–1.58 (m, 1H), 1.59 (dd, *J* = 13.1 and 5.4 Hz, 1H), 1.47 (d, *J* = 15.3, 1H, CH*H*Si), 1.33 (t, *J* = 13.1 Hz, 1H), 1.25–1.23 (m, 1H), 1.12 (s, 3H), 0.82 (s, 3H), 0.78 (d, *J* = 6.8 Hz, 3H), 0.48 (d, *J* = 6.8 Hz, 3H), 0.12 (s, 3H, CH_3_-Si), −0.10 (s, 3H, CH_3_-Si); ^13^C NMR (101 MHz, CDCl_3_) δ = 147.3 (C), 141.2 (C), 133.5 (CH), 128.4 (CH), 127.6 (CH), 127.3 (CH), 126.5 (CH), 126.3 (CH), 82.3 (C), 72.1 (CH_2_), 49.4 (CH), 34.0 (CH_2_), 30.6 (C), 27.8 (CH_3_), 25.2 (CH), 23.9 (CH_3_), 23.8 (CH_3_), 17.9 (CH_3_), 15.9 (CH_2_-Si), −1.1 (CH_3_), −1.3 (CH_3_); HRMS (ESI+) *m/z* calcd for C_25_H_37_OSi ([M + H]^+^): 381.2608, found 381.2610.

(**3k**): distinguishable signals: ^1^H NMR (400 MHz, CDCl_3_) δ = 2.00 (d, *J* = 15.2, 1H, C*H*HSi), 1.79–1.73 (m, 1H), 1.31(d, *J* = 15.2, 1H, CH*H*Si), 1.60–1.58 (m, 1H), 1.15 (s, 3H), 1.07–0.92 (m, 2H), 0.82 (d, *J* = 6.7 Hz, 3H), 0.81 (s, 3H), 0.10 (s, 3H, CH_3_-Si), −0.18 (s, 3H, CH_3_-Si), −0.26 (d, *J* = 6.7 Hz, 3H); ^13^C NMR (101 MHz, CDCl_3_) δ = 133.4 (CH), 128.3 (CH), 127.5 (CH), 127.4 (CH), 126.4 (CH), 82.1 (C), 70.2 (CH_2_), 50.6 (CH), 32.1 (C), 31.9 (CH_2_), 29.6 (CH_3_), 26.6 (CH_2_-Si), 25.9 (CH), 24.9 (CH_3_), 24.3 (CH_3_), 16.1 (CH_3_), −1.5 (CH_3_), −1.9 (CH_3_).

### 3.12. 5,5-Diethyl-3-Methyl-2-Dimethylphenylsilylmethyl-2-Phenyl-Tetrahydropyrans ***2l*** and ***3l***

Chromatography gave tetrahydrofurans **2l** and **3l** as a mixture. Colourless oil (73%).

(**2l**)*:* ^1^H NMR (400 MHz, CDCl_3_) δ = 7.51–7.18 (m, 10H), 3.31 (d, *J* = 11.8, 1H), 3.18 (dd, *J* = 11.8 and 2.2 Hz, 1H), 1.73 (d, *J* = 15.5, 1H, C*H*HSi), 1.70–1.60 (m, 3H), 1.42 (d, *J* = 15.5, 1H, CH*H*Si), 1.41–1.33 (m, 1H), 1.16–1.08 (m, 3H), 0.78 (t, *J* = 7.4 Hz, 3H,), 0.74 (t, *J* = 7.3 Hz, 3H), 0.67 (d, *J* = 6.8 Hz, 3H,), 0.13 (s, 3H, CH_3_-Si), −0.13 (s, 3H, CH_3_-Si); ^13^C NMR (101 MHz, CDCl_3_) δ = 147.7 (C), 141.1 (C), 133.4 (CH), 128.5 (CH), 127.6 (CH), 127.4 (CH), 126.2 (CH), 126.1 (CH), 81.7 (C), 69.0 (CH_2_), 38.3 (CH), 38.1 (CH_2_), 35.4 (C), 28.6 (CH_2_), 23.8 (CH_2_), 17.3 (CH_3_), 13.9 (CH_2_-Si), 7.5 (CH_3_), 7.0 (CH_3_), −1.4 (CH_3_), −1.5 (CH_3_); HRMS (ESI+) *m/z* calcd for C_25_H_36_NaOSi ([M + Na]^+^): 406.2428, found 403.2436.

(**3l**): distinguishable signals: ^13^C NMR (101 MHz, CDCl_3_) δ = 144.2 (C), 128.3 (CH), 127.6 (CH), 127.5 (CH), 127.2 (CH), 68.6 (CH_2_), 38.5 (CH), 36.7 (CH_2_), 28.9 (CH_2_), 28.5 (CH_2_), 26.4 (CH_2_), 18.9 (CH_3_), 14.0 (CH_3_), 7.6 (CH_3_), −1.6 (CH_3_), −1.9 (CH_3_).

### 3.13. Procedure for the Fleming-Tamao Oxidation of Silyl Tetrahydropyran ***2d***

Mercuric acetate (0.466 mmol, 1.5 eq) was added to a solution of **2d** (0.311 mmol) in peracetic acid (35–40% solution in dilute acetic acid; 2 mL) and the mixture was stirred for 3 h at room temperature. Toluene (6 mL) was added and the mixture of solvents was evaporated under reduced pressure. The residue was taken up in ether, filtered and evaporated under reduced pressure. Purification by flash column chromatography (hexane/EtOAc, 3:1 to pure EtOAc) yielded **3d** (0.186 mmol, 60%) as a white viscous liquid (melting point could not be measured). Rf = 0.4 (silica, hexane/EtOAc, 4:1).

### 3.14. Trans-2-Hydroxymethyl-3-Butyl-5,5-Dimethyl-Tetrahydropyran *(**3d**)*

^1^H NMR (400 MHz, CDCl_3_) δ = 3.80 (dd, *J* = 11.4, 2.8 Hz, 1H), 3.56 (dd, *J* = 11.4, 7.1 Hz, 1H), 3.48 (dd, *J* = 10.9, 2.5 Hz, 1H), 3.12 (d, *J* = 10.9 Hz, 1H), 3.04–2.95 (m, 1H), 2.10–1.97 (brs, 1H, OH), 1.65–1.61 (m, 1H), 1.61–1.58 (m, 1H), 1.37–1.13 (m, 5H), 1.01 (s, 3H), 0.98–0.95 (m, 1H), 0.94–0.90 (m, 1H), 0.88 (t, *J* = 7.1 Hz, 3H), 0.82 (s, 3H); ^13^C NMR (101 MHz, CDCl_3_) δ = 82.4 (CH), 77.7 (CH_2_), 63.9 (CH_2_OH), 42.7 (CH_2_), 32.6 (CH, Bu), 31.2 (CH_2_), 30.9 (C), 28.3 (CH_2_), 27.2 (CH_3_), 24.0 (CH_3_), 22.9 (CH_2_), 14.0 (CH_3_, Bu); HRMS (ESI+) *m/z* calcd for C_12_H_24_NaO_2_ ([M + Na]^+^): 223.1669, found 223.1667.

## 4. Conclusions

In conclusion, a general and efficient methodology for the synthesis of tetrahydropyrans by the acid-mediated cyclizations of vinylsilyl alcohols is described. The reaction leading to polysubstituted tetrahydropyrans is highly stereoselective when R^4^ is either H or alkyl group. Worthy of note, quaternary centres adjacent to the oxygen can be formed through the process. The formation of a single diastereoisomer in these cases (THP with the phenyldimethylsilylmethyl group anti to the C-3 substituent) seems to be a consequence of an unfavourable 1,3-diaxial interaction in the alternative reactive conformation. Moreover, the presence of the silyl group bonded to the alkenyl moiety seems to be needed for the cyclization to take place. Further transformation of the silylated tetrahydropyrans thus obtained into the corresponding hydroxymethyl tetrahydropyrans opens an attractive route for the synthesis of marine drugs analogues.

## Figures and Tables

**Table 1:**
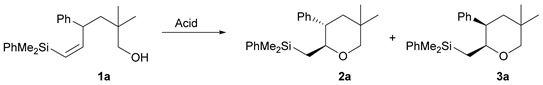
Optimization of the acid-mediated cyclization.

Entry	Acid ^1^	Temperature (°C)	Solvent	Ratio 2a/3a ^2^	Product, Yield
1	TMSOTf	−78	CH_2_Cl_2_		Complex mixture
2	TMSOTf	−78	Et_2_O		Complex mixture
3	TiCl_4_	−78	CH_2_Cl_2_		Complex mixture
4	ZnCl_2_	−78	CH_2_Cl_2_		n.r. ^3^
5	SiO_2_	r. t.	AcOEt		n.r. ^3^
6	BF_3_·OEt_2_	−78	CH_2_Cl_2_		n.r. ^3^
7	BF_3_·OEt_2_	0	CH_2_Cl_2_	67:33	**2a** + **3a** (69%)
8	SnCl_4_	−78	CH_2_Cl_2_	50:50	**2a** + **3a** (63%)
9	CSA	reflux	CH_2_Cl_2_		n.r. ^3^
10	p-TsOH	reflux	CH_2_Cl_2_	>95:5	**2a** (77%)

^1^ 1.0 equiv. of acid is used in every example. ^2^ The ratio of isomers **2a** and **3a** was determined by ^1^H-NMR analysis. ^3^ n.r. stands for no reaction.

**Table 2:**
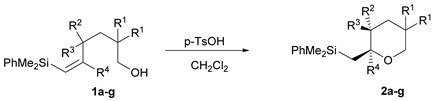
Cyclization of vinylsilyl alcohols **1a**–**g**.

Entry	R^1^	R^2^	R^3^	R^4^	Time (h) ^1^	*dr* ^2^	Yield (%) ^3^
1	Me	Ph	H	H	1	˃95:5	**2a** (77)
2	Me	Ph	H	H	4	˃95:5	**2a** (62) ^4^
3	Me	Ph	H	H	4	˃95:5	**2a** (67) ^5^
4	Me	H	H	H	1		**2b** (71)
5	Me	Me	Me	H	1		**2c** (70)
6	Me	Bu	H	H	1	>95:5	**2d** (73)
7	Me	^i^Pr	H	H	1	>95:5	**2e** (79)
8	Me	Ph	H	^n^Pr	1	>95:5	**2f** (71)
9	Et	Me	H	^n^Pr	1	>95:5	**2g** (70)
10	Me	Ph	H	SiMe_3_	3		n.r. ^6^

^1^ All the reactions were run at reflux temperature except where indicated. ^2^ The relative stereochemistry of tetrahydropyrans **2** was assigned based on the 1D-NOE experiments (in every compound 1D-crosspeak was found between C*H*_2_-Si and C*H*-R^2^). ^3^ Yields over two steps from the corresponding epoxides precursor of **1**. ^4^ The reaction did not go to completion when 0.5 equiv. of pTsOH were used. ^5^ The reaction was run at r.t. and partial desilylation of the final THP was observed. ^6^ n.r. stands for no reaction.

**Table 3:**
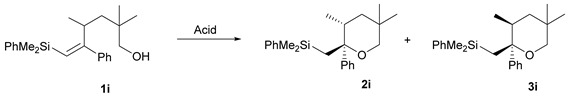
Optimization of the cyclization of aryl substituted vinylsilyl alcohol **1i**.

Entry	Acid ^1^	Temperature (°C)	Solvent	Time (min)	Product (yield)
1	BF_3_·OEt_2_	0	CH_2_Cl_2_	60	Desilylated THP ^2^ (52%)
2	SnCl_4_	−78	CH_2_Cl_2_	90	Desilylated THP (48%)
3	p-TsOH	reflux	CH_2_Cl_2_	60	Desilylated THP (69%)
4	p-TsOH	r.t.	CH_2_Cl_2_	30	**2i** + **3i** + Desilylated THP (55%) ^3^
5	p-TsOH	0	CH_2_Cl_2_	10	**2i** + **3i** (71%) ^4^
6	CSA	r.t.	CH_2_Cl_2_	30	**2i** + **3i** (70%) ^4^
7	CSA	0	CH_2_Cl_2_	60	**2i** + **3i** (69%) ^4^

^1^ 1.0 equiv. of acid is used in every example. ^2^ Desilylated THP accounts for 2,3,5,5-tetramethyl-2-phenyl-tetrahydropyran. ^3^ A 2:1 mixture of the silylated and desilylated tetrahydropyrans was obtained. ^4^ A 78:22 mixture of **2i**:**3i** was obtained.

**Table 4:**
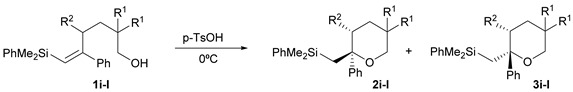
Cyclization of arylsubstituted vinylsilyl alcohols **1i**–**l**.

Entry	R^1^	R^2^	Time (min)	Product (Ratio 2:3)	Yield (%) ^1^
1	Me	Me	10	78:22	**2i** + **3i** (71)
2	Me	Ph	10	79:21	**2j** + **3j** (65)
3	Me	^i^Pr	10	91:9	**2k** + **3k** (72)
4	Et	Me	10	86:14	**2l** + **3l** (73)

^1^ Yields over two steps from the epoxide precursor of **1**.

## Supplementary Materials

Copies of ^1^H-NMR and ^13^C-NMR are available online at http://www.mdpi.com/1660-3397/16/11/421/s1.
