# Supplementary material for: Synthesis of Polysubstituted Tetrahydropyrans by Stereoselective Hydroalkoxylation of Silyl Alkenols: En Route to Tetrahydropyranyl Marine Analogues
Source: Mar Drugs. 2018 Nov 1;16(11):421. doi: 10.3390/md16110421 (PMC6265778; doi:10.3390/md16110421)
Supplement: Supplementary file 1 [file marinedrugs-16-00421-s001.pdf]

Supporting Information

Synthesis of polisubstituted tetrahydropyrans by stereoselective hydroalkoxylation of silyl alkenols: en route to tetrahydropyranyl marine analogs

C. Díez-Poza, P. Val, F. J. Pulido and A. Barbero*

Synthesis of polisubstituted tetrahydropyrans by stereoselective hydroalkoxylation of silyl alkenols: en route to tetrahydropyranyl marine analogs

C. Díez-Poza, P. Val, F. J. Pulido and A. Barbero*

Department of Organic Chemistry, University of Valladolid, Campus Miguel Delibes, 47011 Valladolid (SPAIN).

Contents

1. **General Information**
2. **^{1}H and ^{13}C NMR Spectra**

1. General methods

Unless otherwise noted, all reagents were obtained from commercial suppliers as reagent grade and used without further purification. Analytically pure solvents were purchased from commercial suppliers and used without further purification. Tetrahydrofuran, diethyl ether and dichloromethane were dried by standard methods (dichloromethane was freshly distilled from CaH_2, tetrahydrofuran and diethyl ether were dried with preactivated molecular sieves. All experiments were performed in oven dried glassware under an atmosphere of nitrogen using standard syringe techniques, except where otherwise noted. Yields refer to chromatographically and spectroscopically pure compounds, unless otherwise stated.

NMR spectra were recorded using Agilent MR 400 MHz and Agilent VNMRS 500 MHz with cold probe spectrometers, at room temperature (25ºC). High-resolution mass spectra (HRMS) were measured using a UPLC-MS system (UPLC: Waters ACQUITY H-class UPLC; MS: Bruker Maxis Impact) by electrospray ionization (ESI positive and negative).

The relative stereochemistry of tetrahydropyranes was assigned based on the 1D-NOE or NOESY experiments, considering the signals corresponding to the interactions highlighted in the figure:

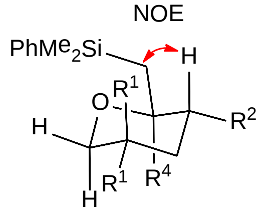

2. **^{1}H and ^{13}C NMR Spectra**

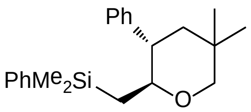

2a

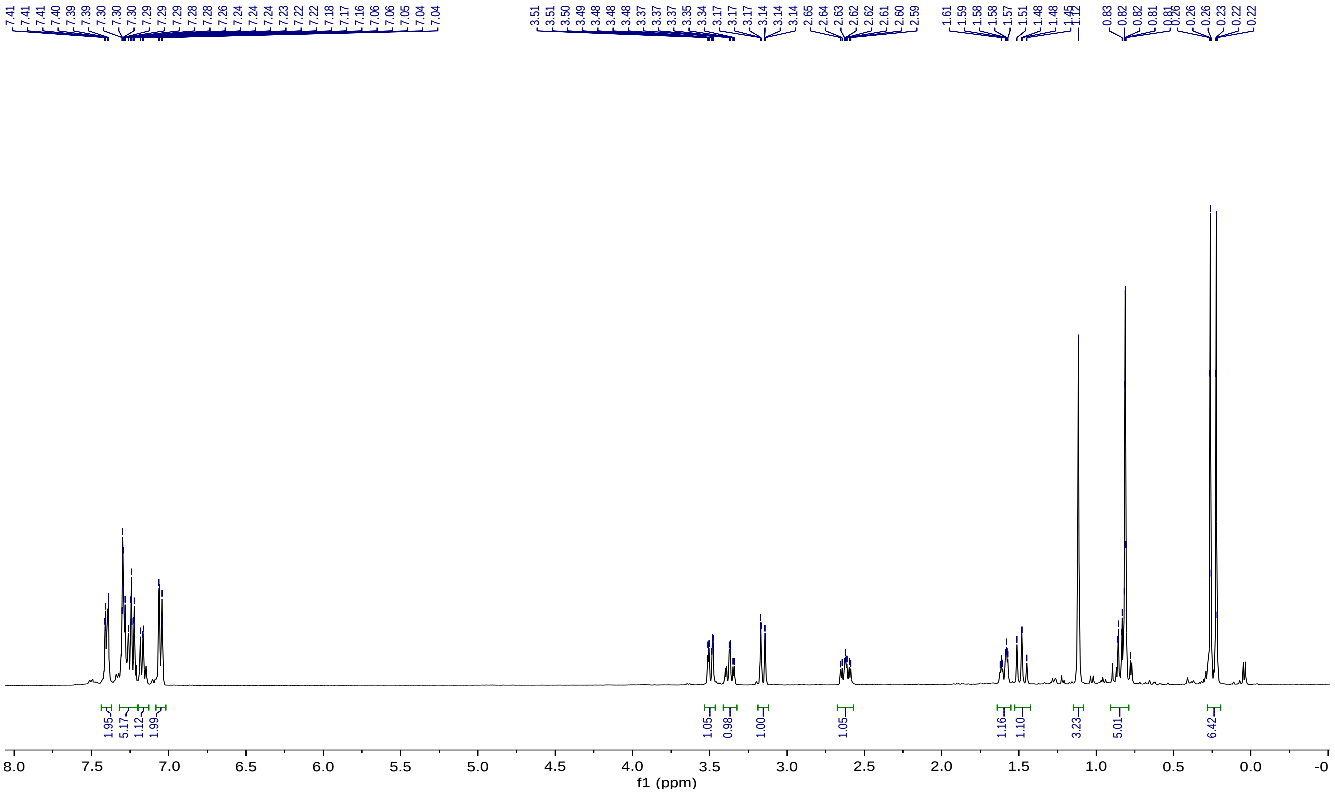

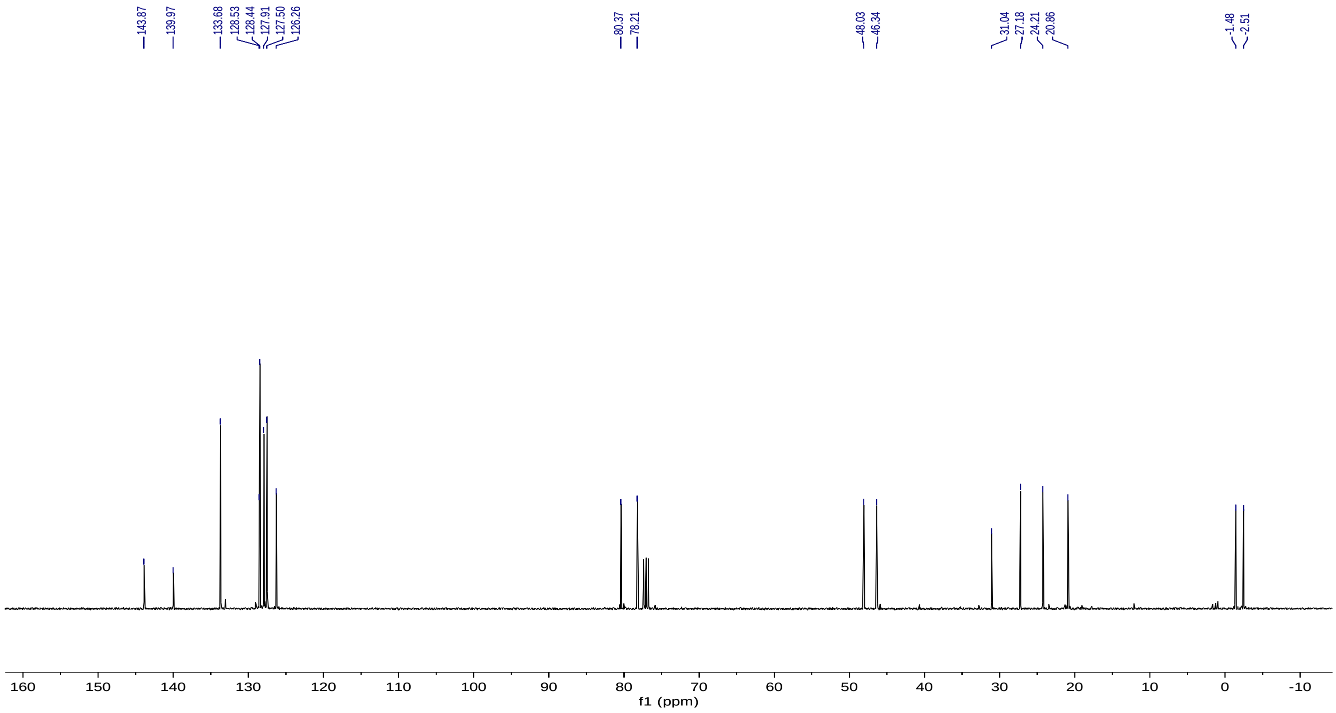

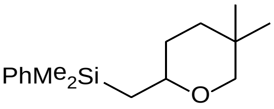
PhMe2Si
O

2b

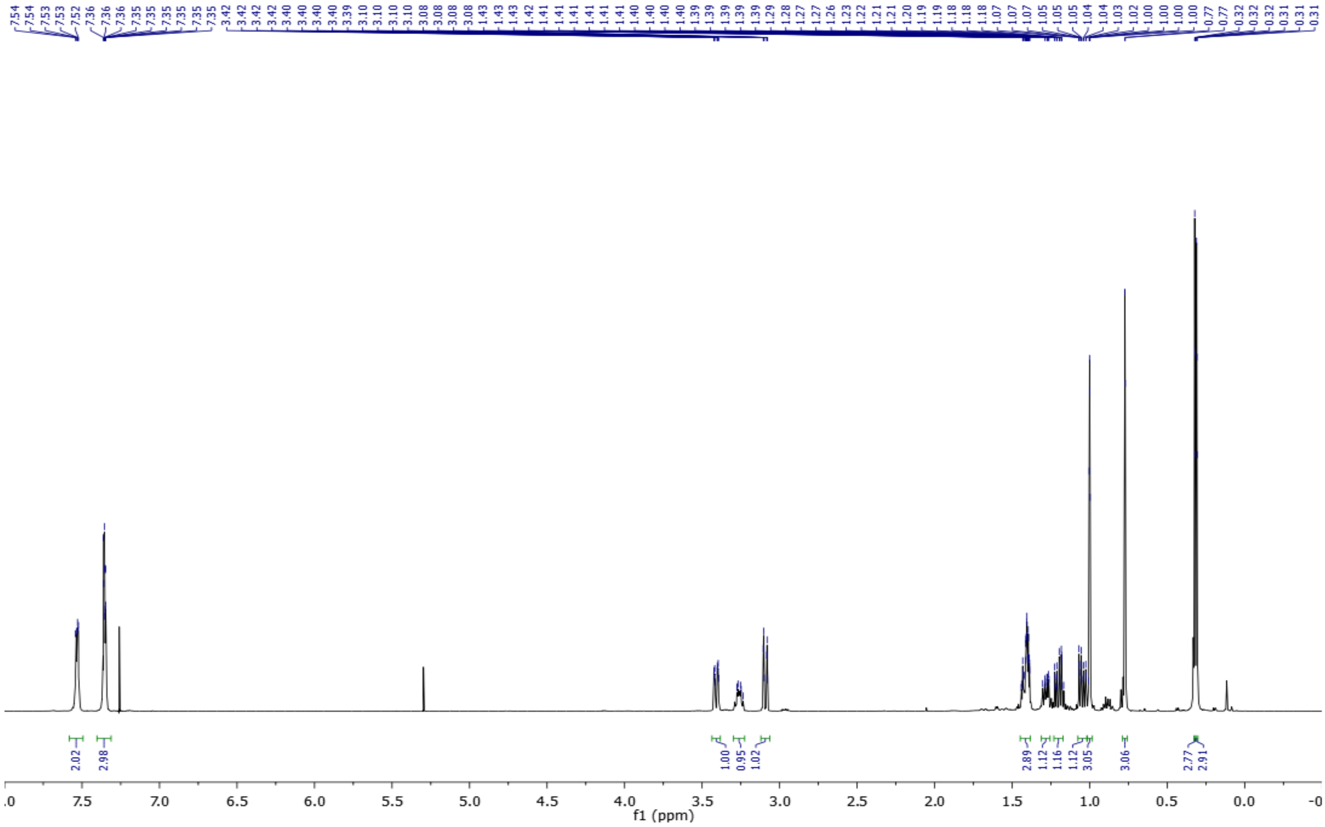
7.54 7.54 7.53 7.53 7.52 7.36 7.36 7.35 7.35 7.35 7.35 7.35 7.35 3.42 3.42 3.42 3.40 3.40 3.40 3.40 3.39 3.10 3.10 3.10 3.10 3.08 3.08 3.08 3.08 1.43 1.43 1.43 1.42 1.41 1.41 1.41 1.41 1.41 1.40 1.40 1.40 1.39 1.39 1.39 1.39 1.39 1.29 1.28 1.27 1.27 1.26 1.23 1.22 1.21 1.21 1.20 1.19 1.19 1.18 1.18 1.18 1.07 1.07 1.05 1.05 1.05 1.04 1.04 1.03 1.02 1.00 1.00 1.00 1.00 0.77 0.77 0.32 0.32 0.31 0.31
2.02 2.98 1.00 0.95 1.02 2.89 1.12 1.16 1.12 3.05 3.06 2.77 2.91
7.5 7.0 6.5 6.0 5.5 5.0 4.5 4.0 3.5 3.0 2.5 2.0 1.5 1.0 0.5 0.0
f1 (ppm)

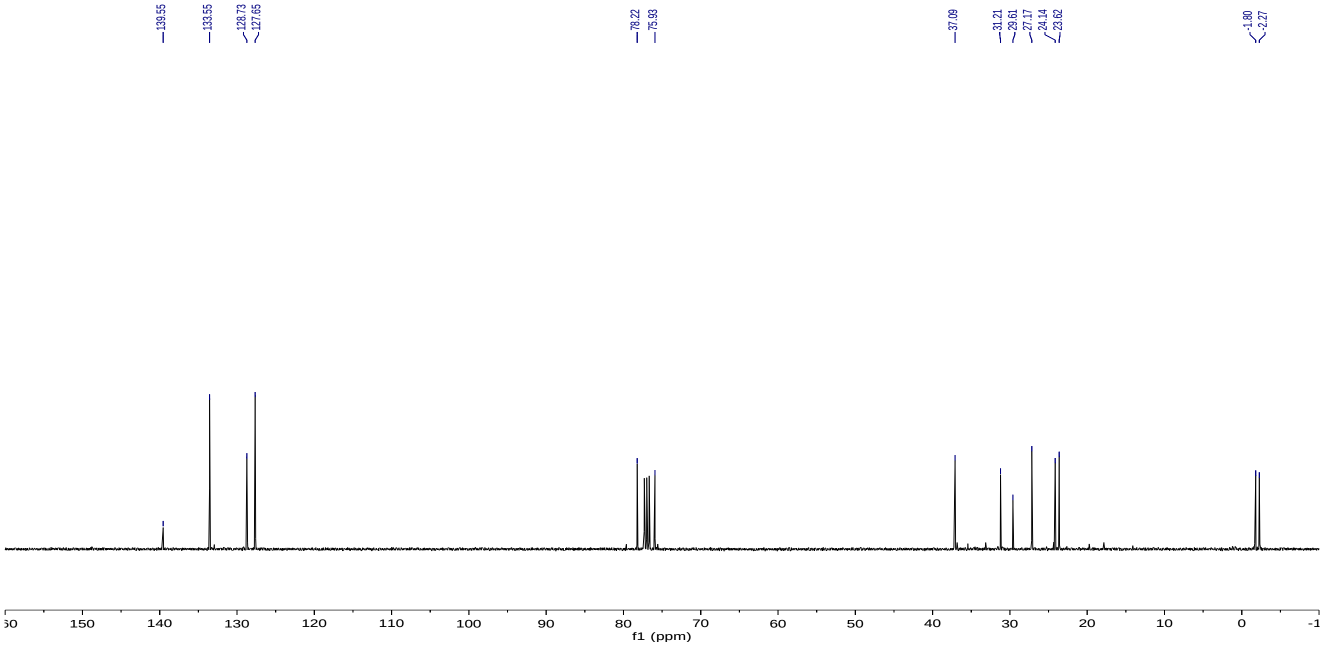
139.55 133.55 128.73 127.65 78.22 75.93 37.09 31.21 29.61 27.17 24.14 23.62 -1.80 -2.27
150 140 130 120 110 100 90 80 70 60 50 40 30 20 10 0
f1 (ppm)

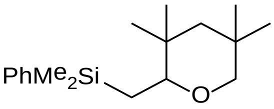

2c

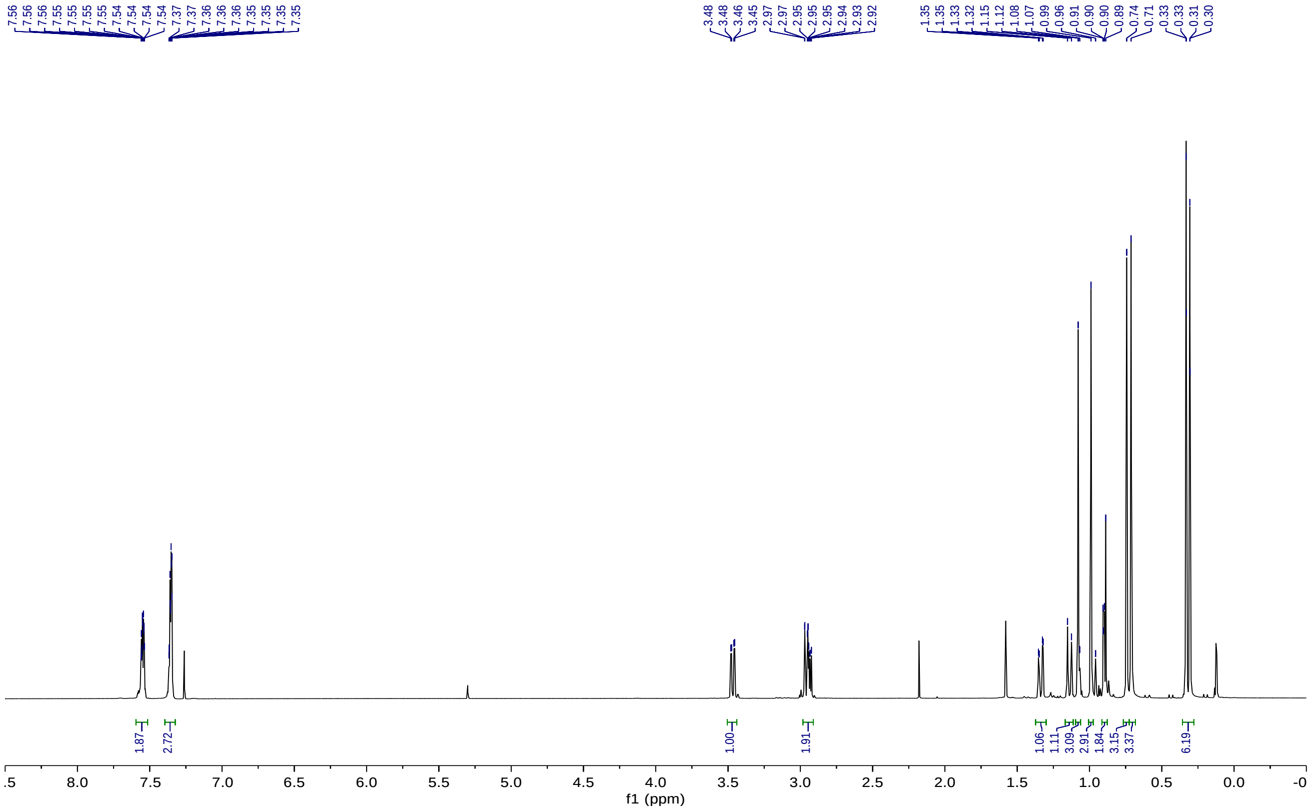

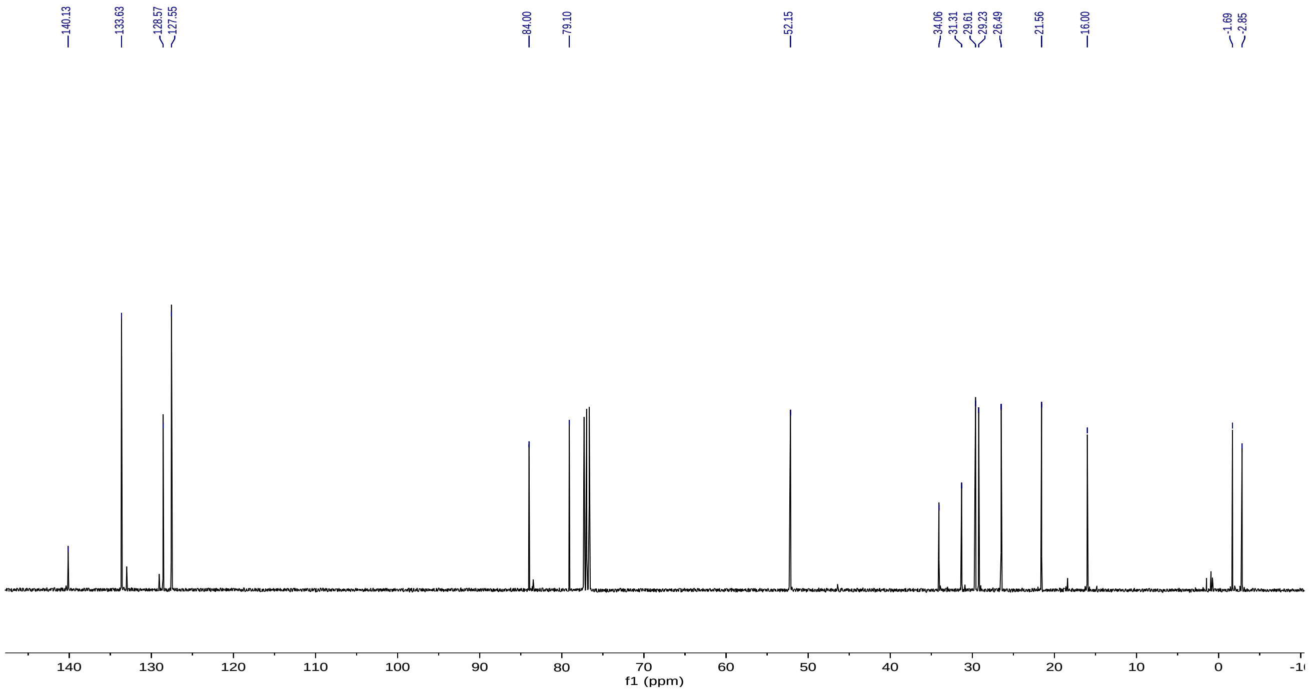

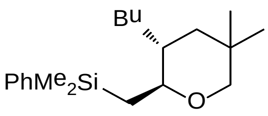

Bu
PhMe2Si
O

2d

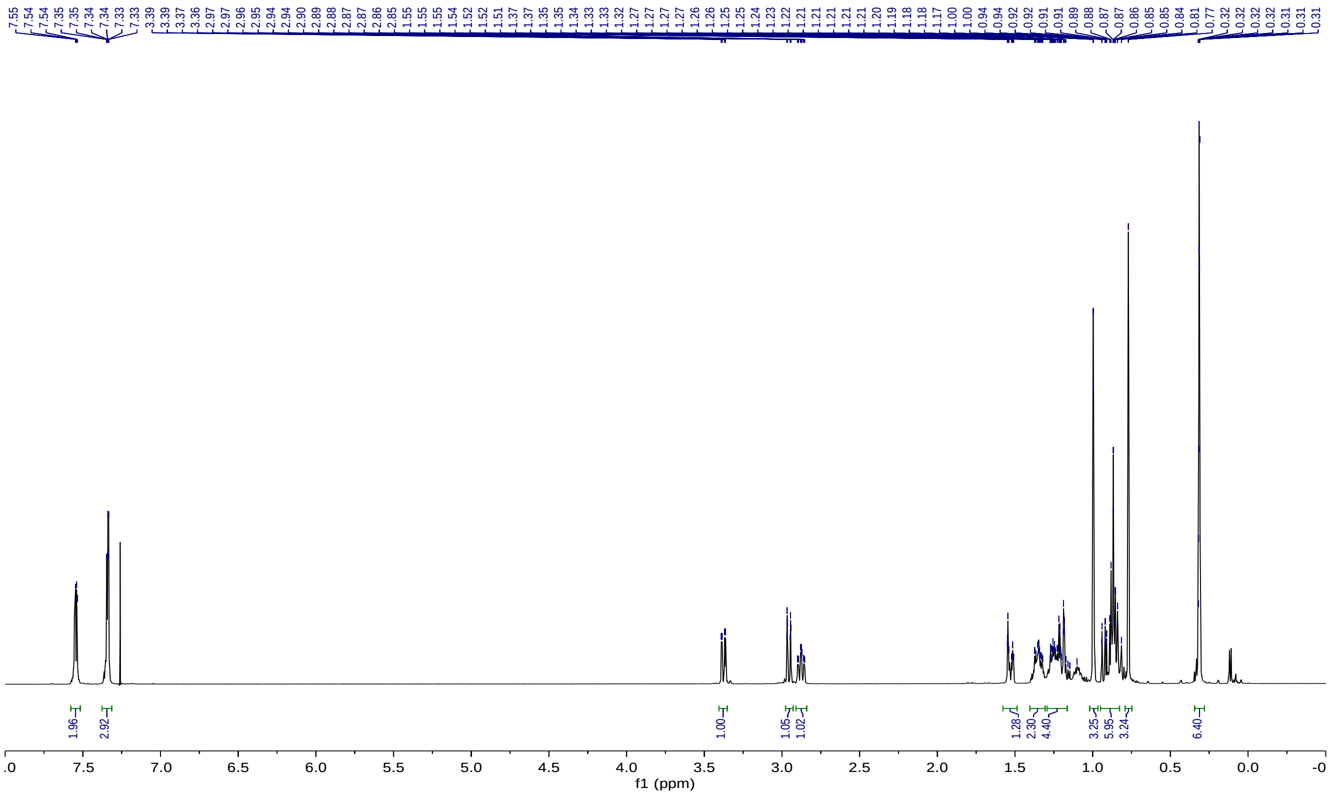

7.55 7.54 7.54 7.35 7.35 7.34 7.34 7.33 7.33 3.39 3.39 3.37 3.36 2.97 2.97 2.96 2.95 2.94 2.94 2.90 2.89 2.88 2.87 2.87 2.86 2.85 1.55 1.55 1.55 1.54 1.52 1.52 1.51 1.37 1.37 1.35 1.35 1.34 1.33 1.33 1.32 1.27 1.27 1.27 1.27 1.26 1.26 1.25 1.25 1.24 1.23 1.22 1.21 1.21 1.21 1.21 1.21 1.20 1.19 1.18 1.18 1.17 1.00 1.00 0.94 0.94 0.92 0.92 0.91 0.91 0.89 0.88 0.87 0.87 0.86 0.85 0.85 0.84 0.81 0.77 0.32 0.32 0.32 0.32 0.31 0.31
1.96 2.92 1.00 1.05 1.02 1.28 2.30 4.40 3.25 5.95 3.24 6.40
f1 (ppm)

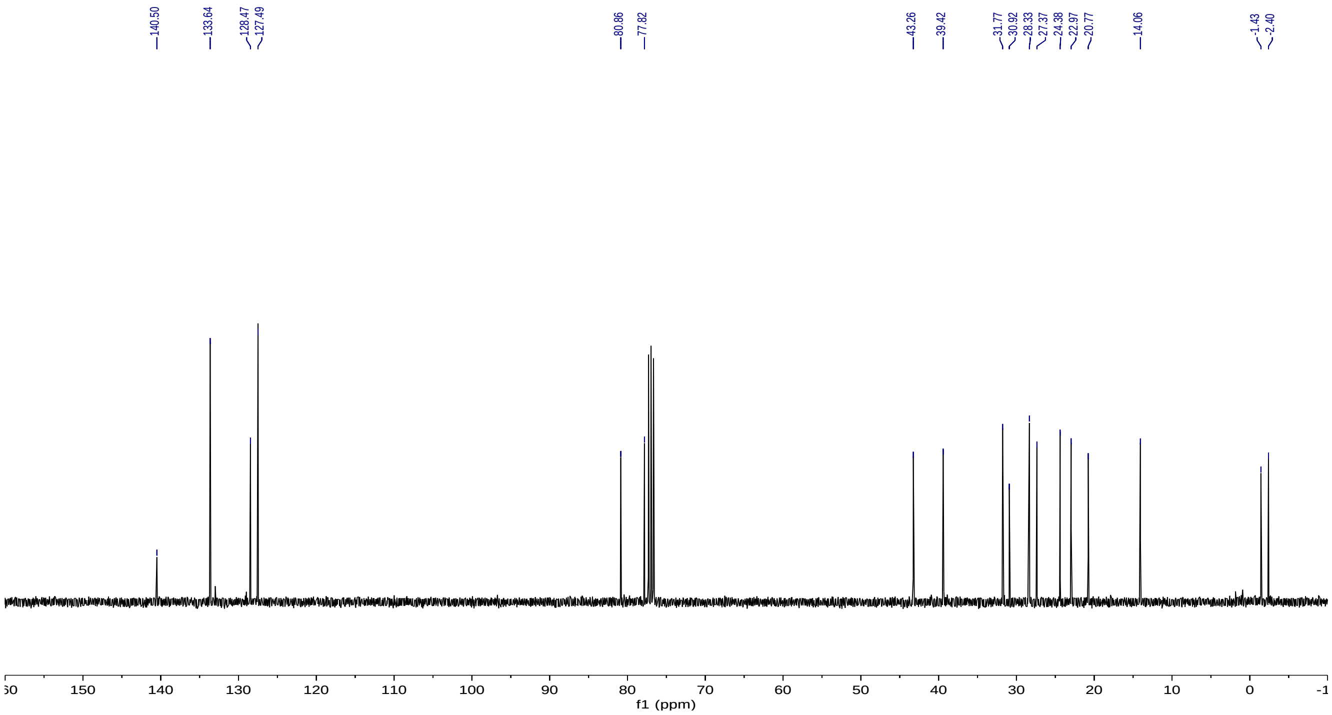

140.50 133.64 128.47 127.49 80.86 77.82 43.26 39.42 31.77 30.92 28.33 27.37 24.38 22.97 20.77 14.06 -1.43 -2.40
f1 (ppm)

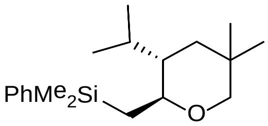

2e

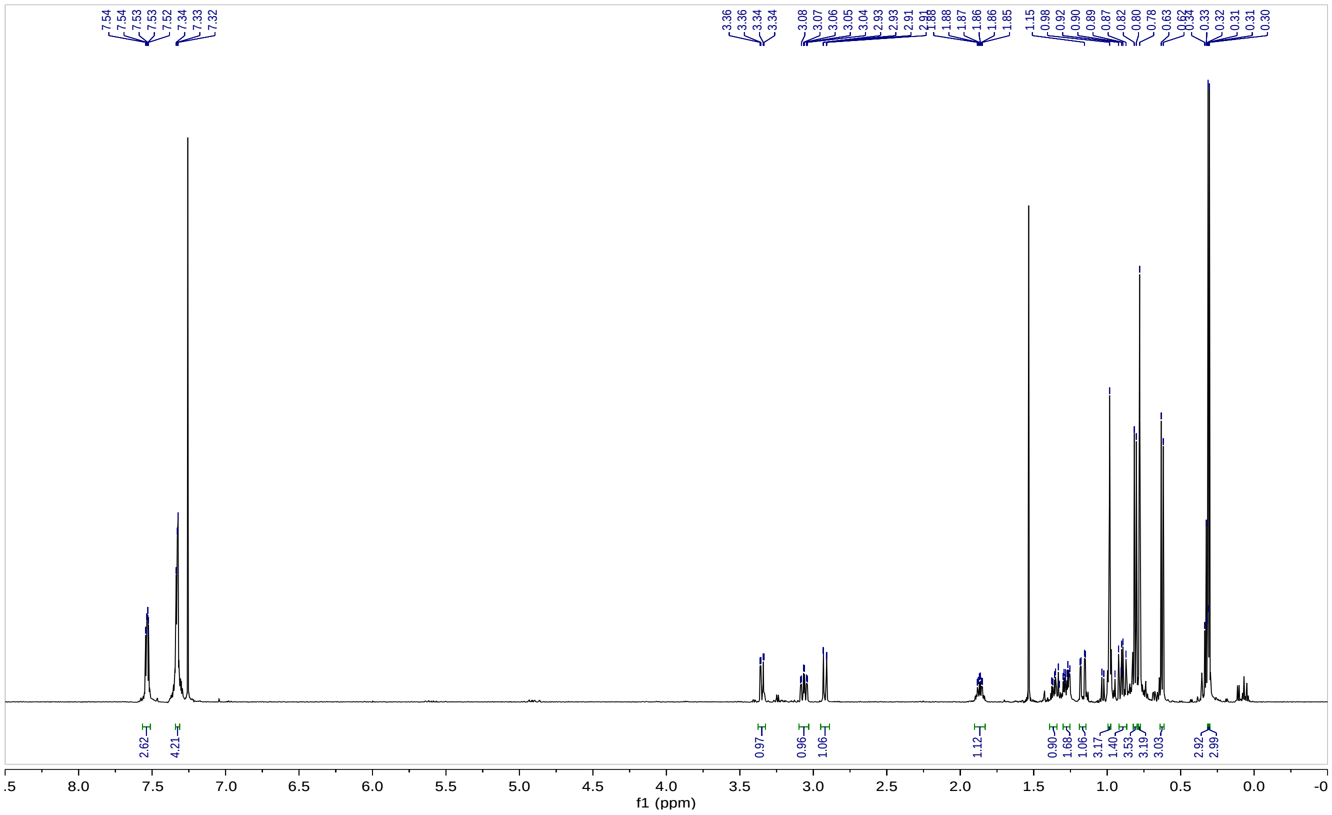

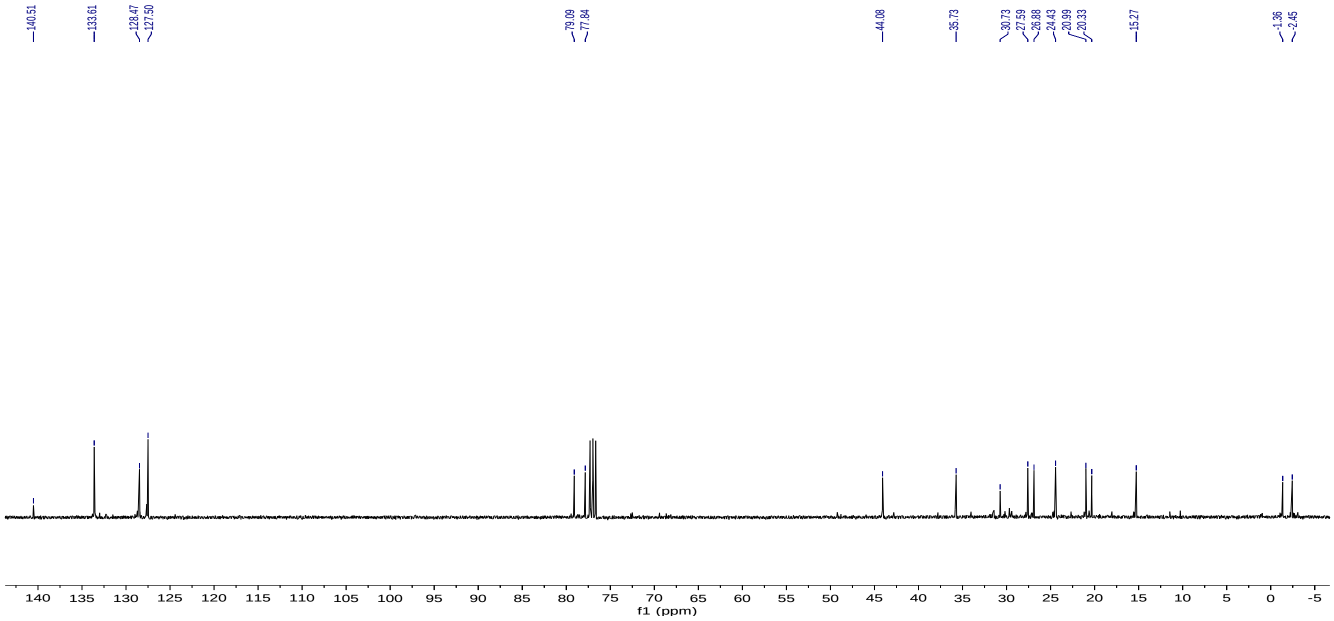

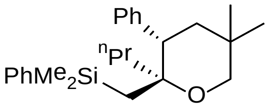

2f

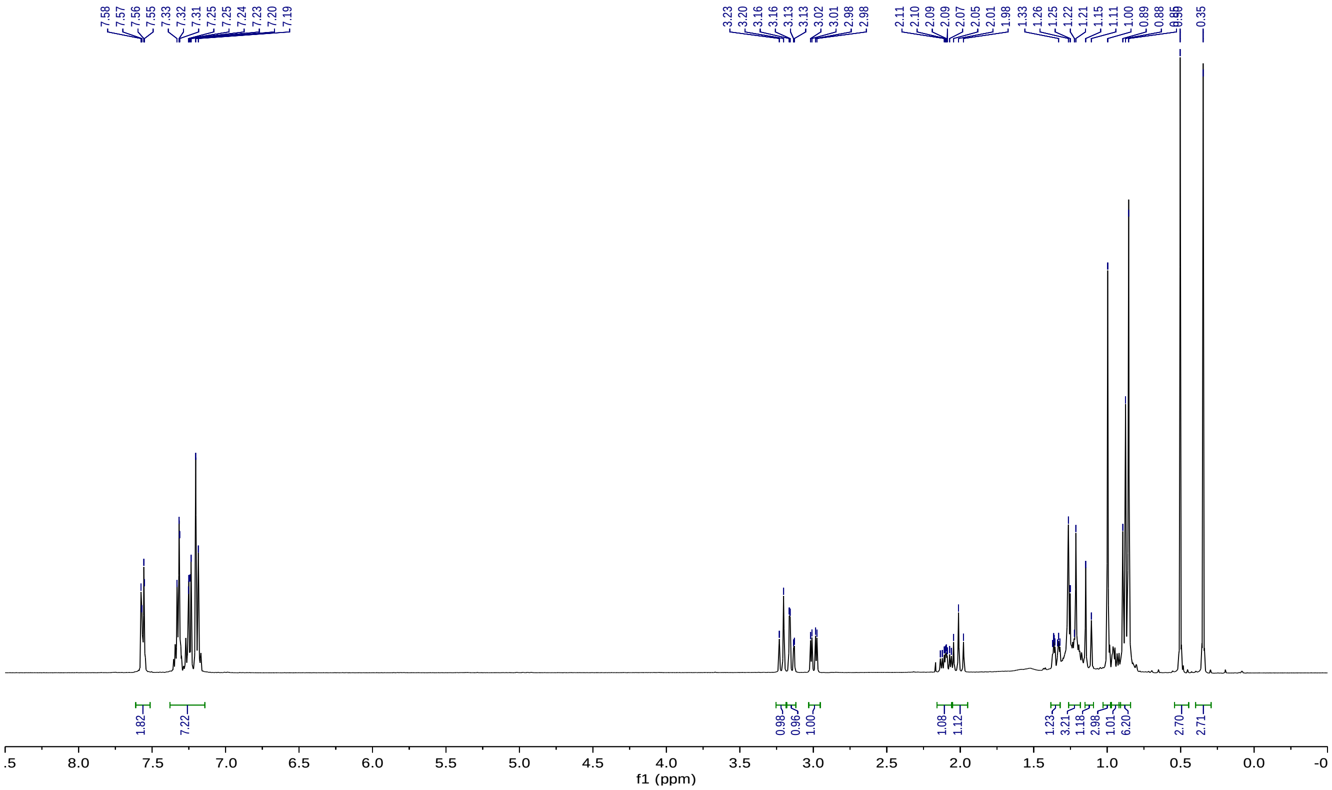

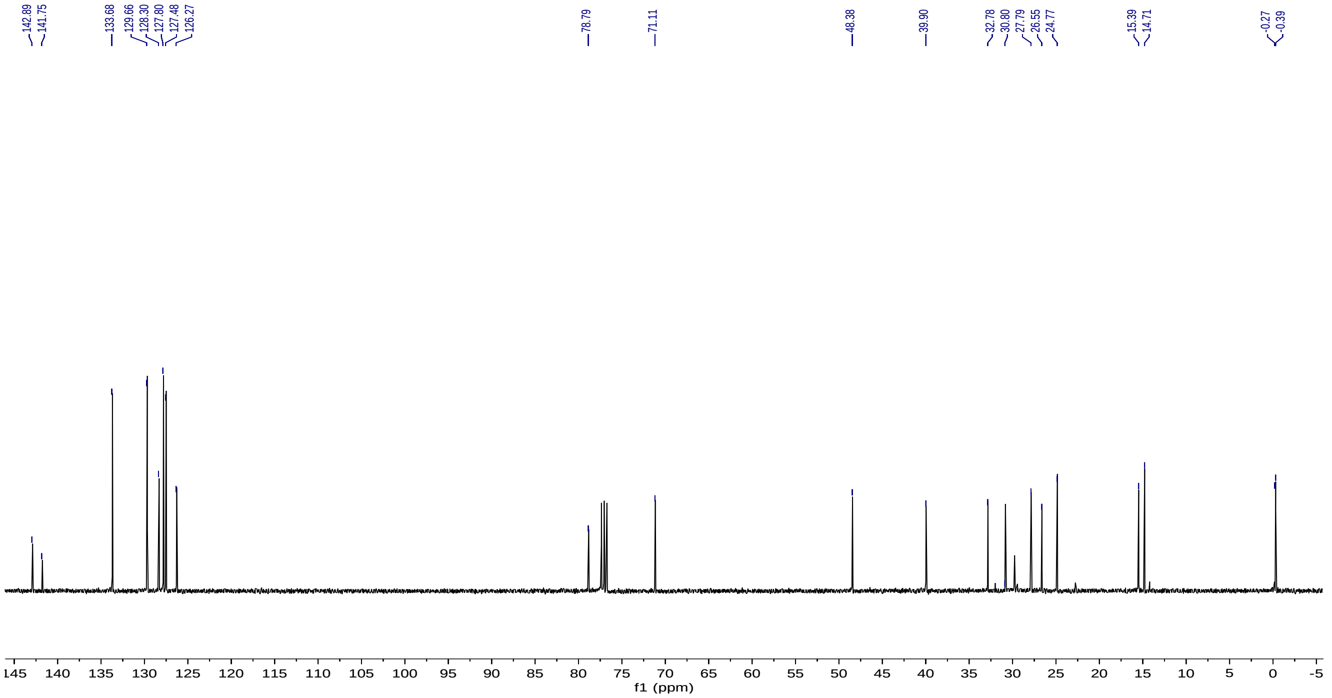

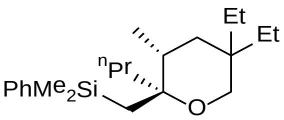
Et
Et
nPr
PhMe2Si
O

2g

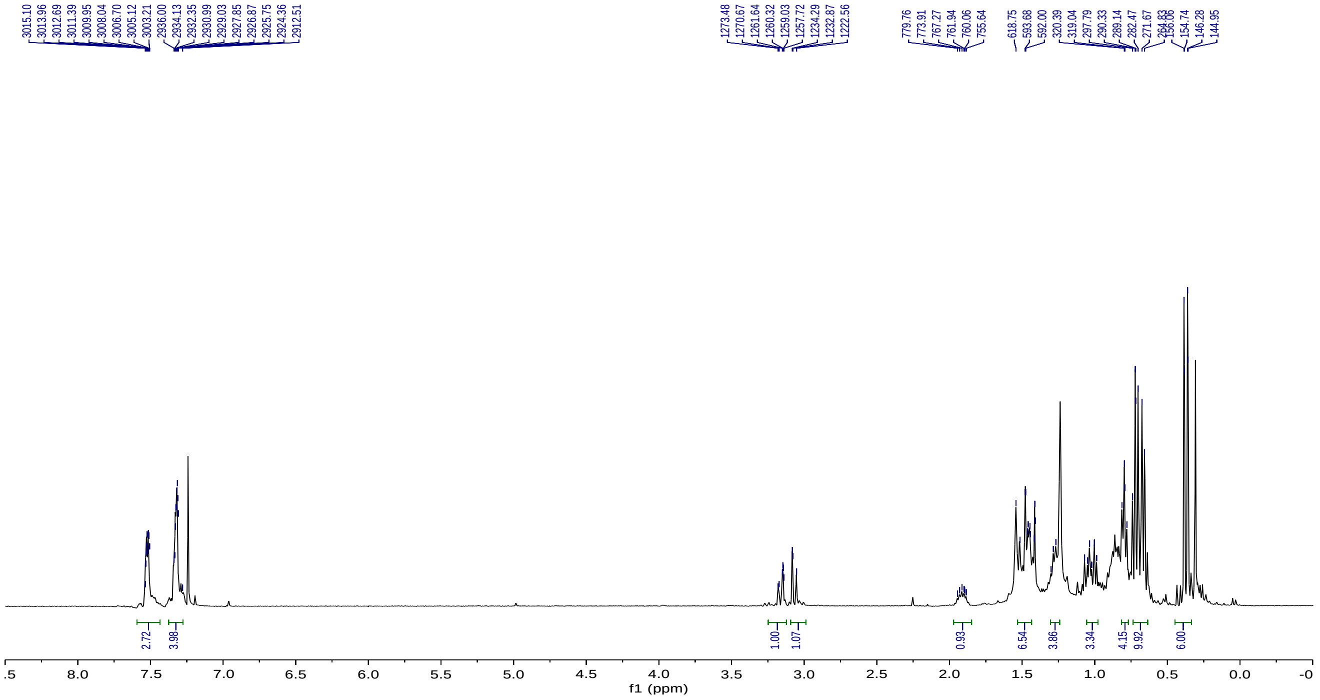
3015.10
3013.96
3012.69
3011.39
3009.95
3008.04
3006.70
3005.12
3003.21
2936.00
2934.13
2932.35
2930.99
2929.03
2927.85
2926.87
2925.75
2924.36
2912.51
1273.48
1270.67
1261.64
1260.32
1259.03
1257.72
1234.29
1232.87
1222.56
779.76
773.91
767.27
761.94
760.06
755.64
618.75
593.68
592.00
320.39
319.04
297.79
290.33
289.14
282.47
271.67
264.83
156.06
154.74
146.28
144.95
2.72
3.98
1.00
1.07
0.93
6.54
3.86
3.34
4.15
9.92
6.00
.5 8.0 7.5 7.0 6.5 6.0 5.5 5.0 4.5 4.0 3.5 3.0 2.5 2.0 1.5 1.0 0.5 0.0 -0
f1 (ppm)

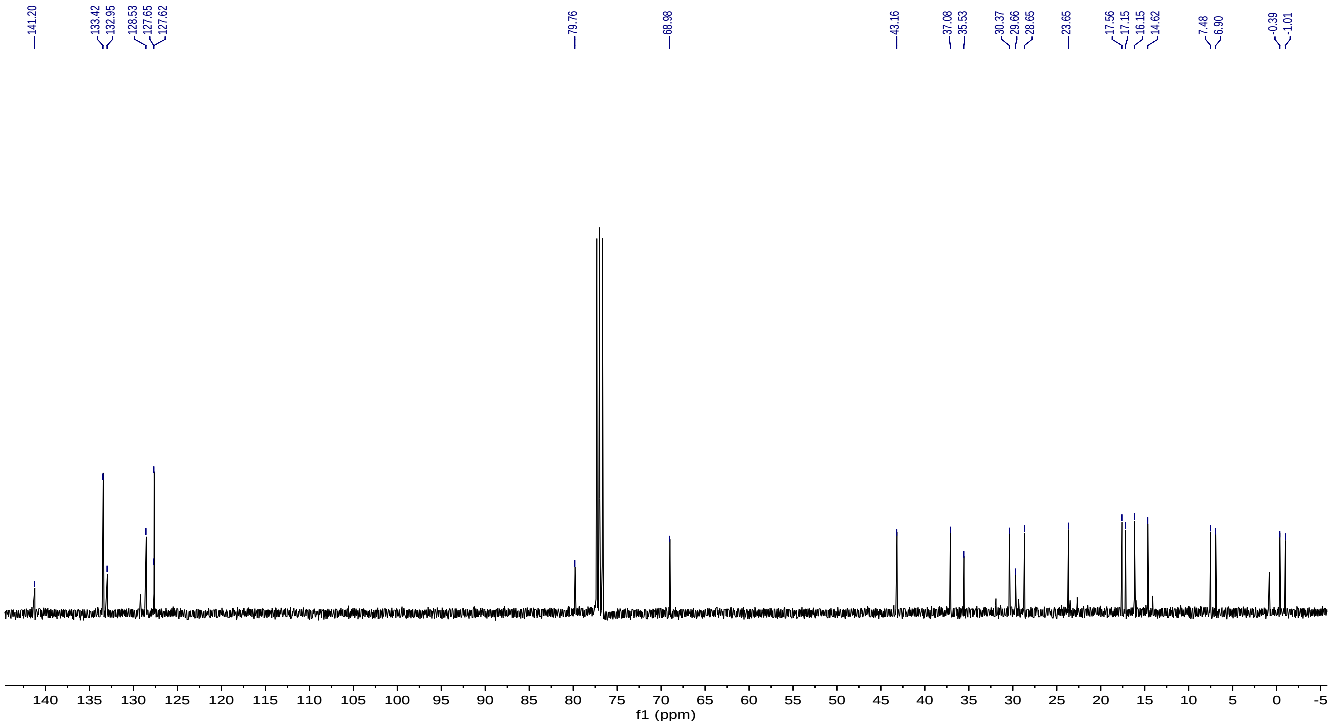
141.20
133.42
132.95
128.53
127.65
127.62
79.76
68.98
43.16
37.08
35.53
30.37
29.66
28.65
23.65
17.56
17.15
16.15
14.62
7.48
6.90
-0.39
-1.01
140 135 130 125 120 115 110 105 100 95 90 85 80 75 70 65 60 55 50 45 40 35 30 25 20 15 10 5 0 -5
f1 (ppm)

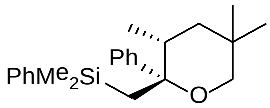

2i major (+ **3i** minor)

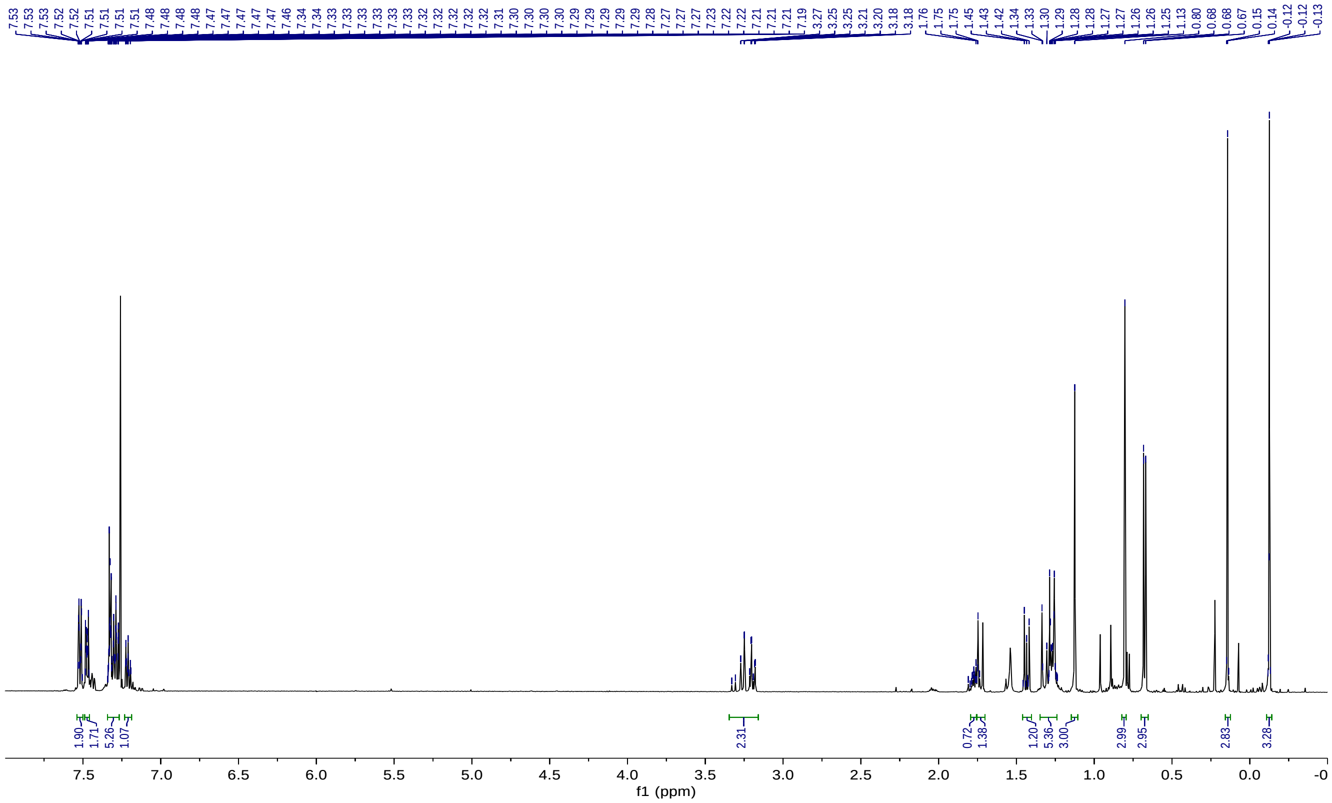

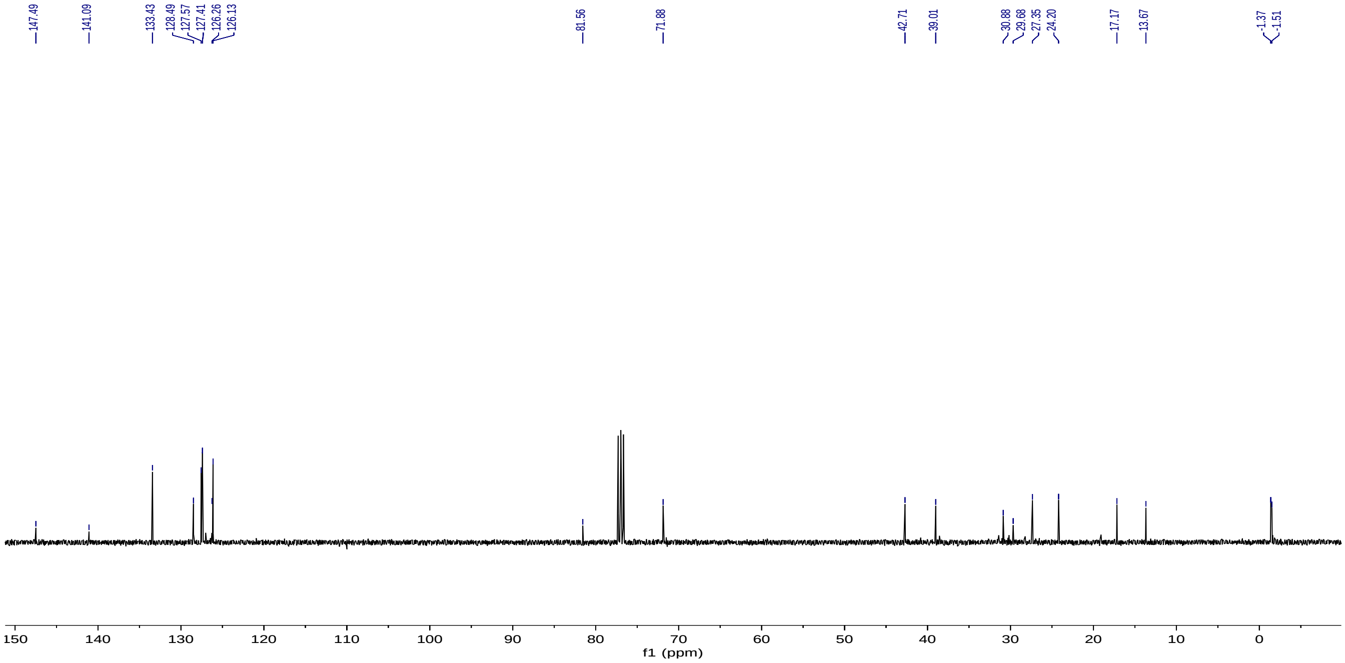

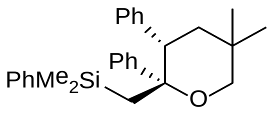

2j major (+ **3j** minor)

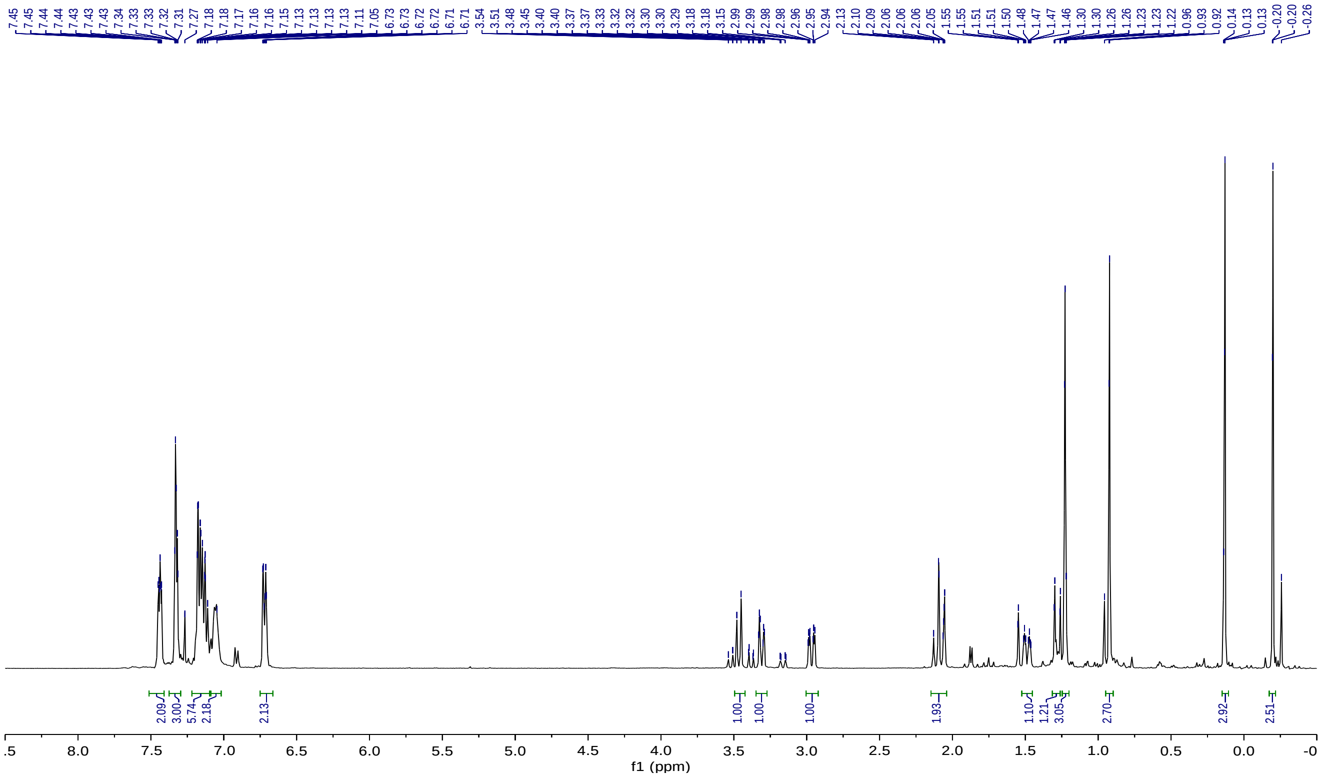

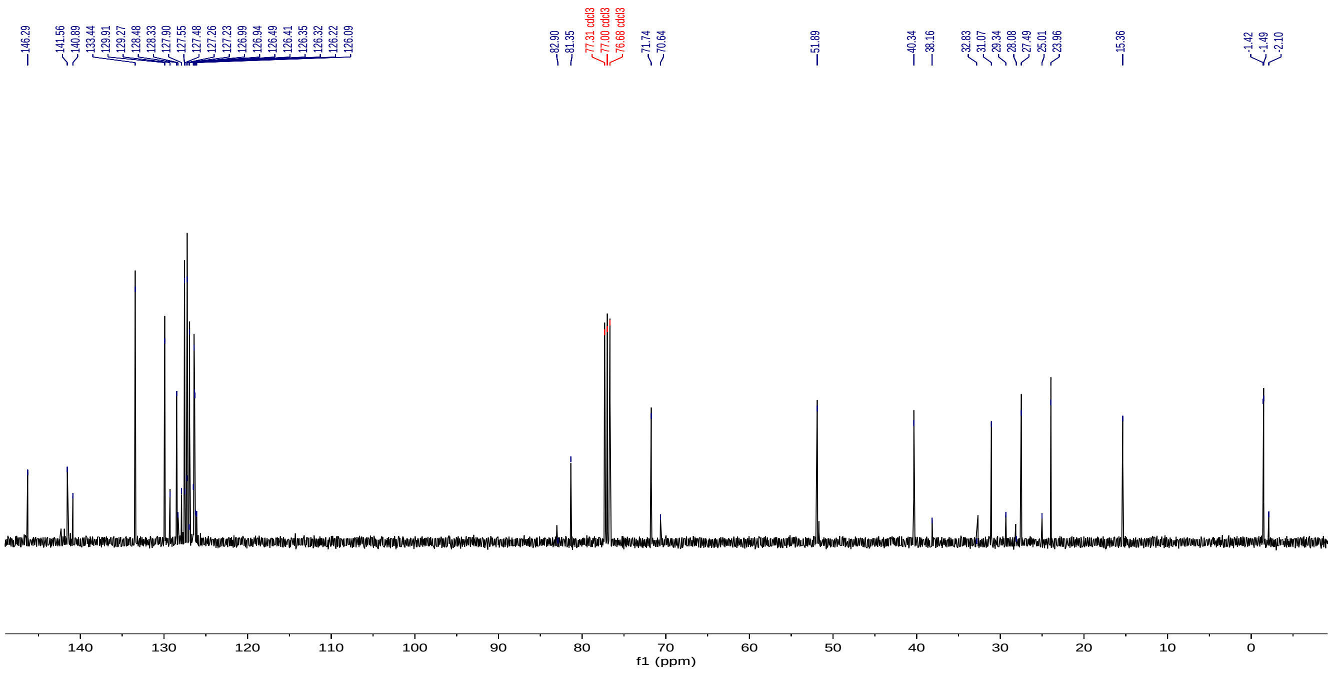

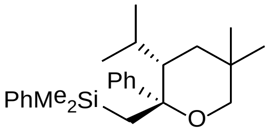
Ph
PhMe2Si
O

2k

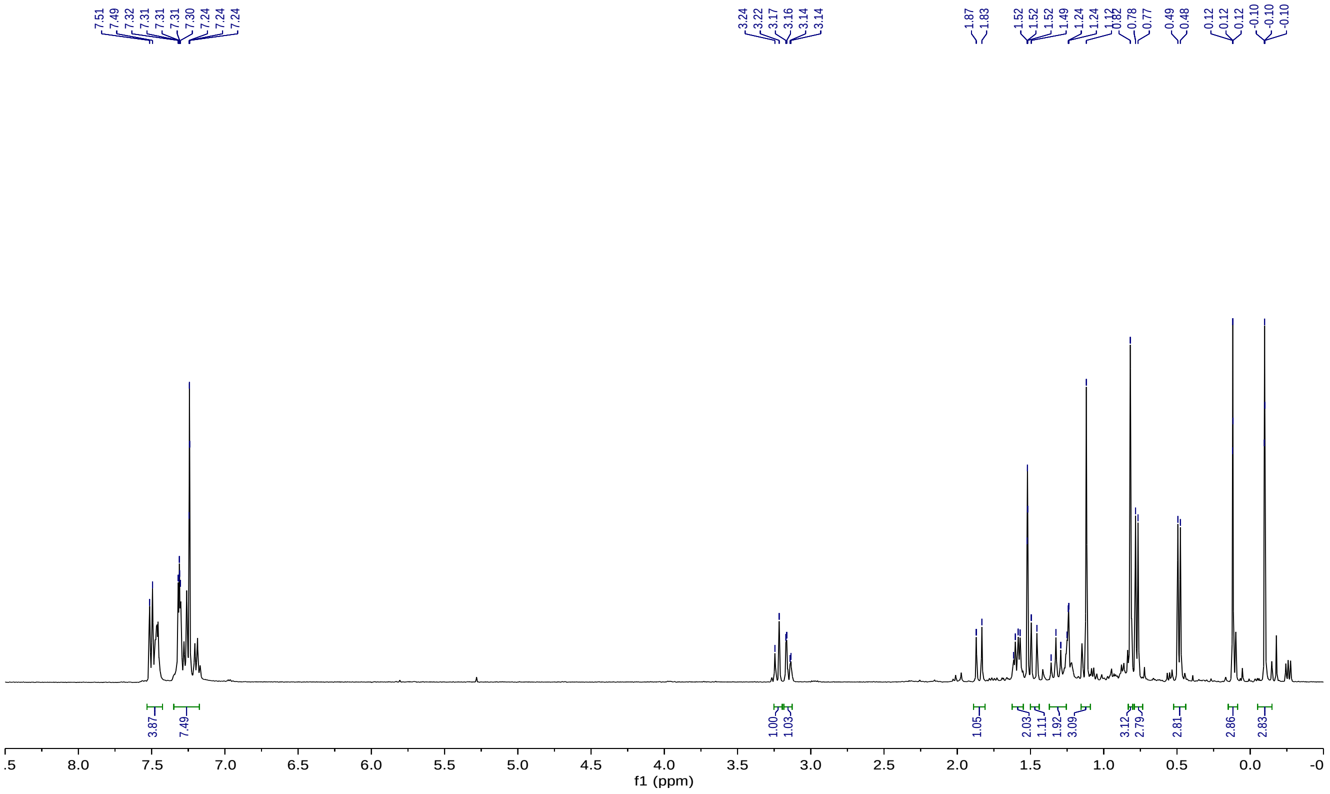
7.51
7.49
7.32
7.31
7.31
7.31
7.30
7.24
7.24
7.24
3.24
3.22
3.17
3.16
3.14
3.14
1.87
1.83
1.52
1.52
1.52
1.49
1.24
1.24
1.12
0.82
0.78
0.77
0.49
0.48
0.12
0.12
0.12
-0.10
-0.10
-0.10
3.87
7.49
1.00
1.03
1.05
2.03
1.11
1.92
3.09
3.12
2.79
2.81
2.86
2.83
f1 (ppm)

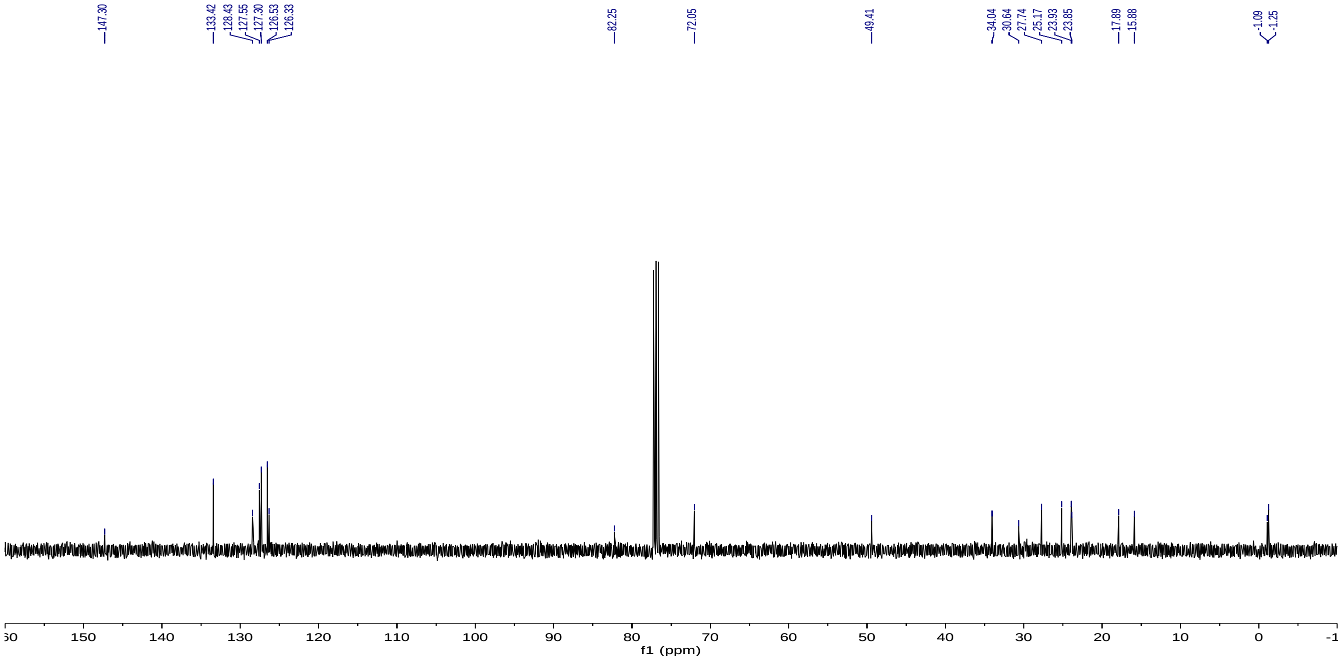
147.30
133.42
128.43
127.55
127.30
126.53
126.33
82.25
72.05
49.41
34.04
30.64
27.74
25.17
23.93
23.85
17.89
15.88
-1.09
-1.25
f1 (ppm)

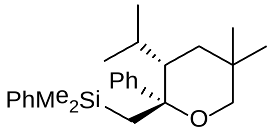

2k major (+ **3k** minor)

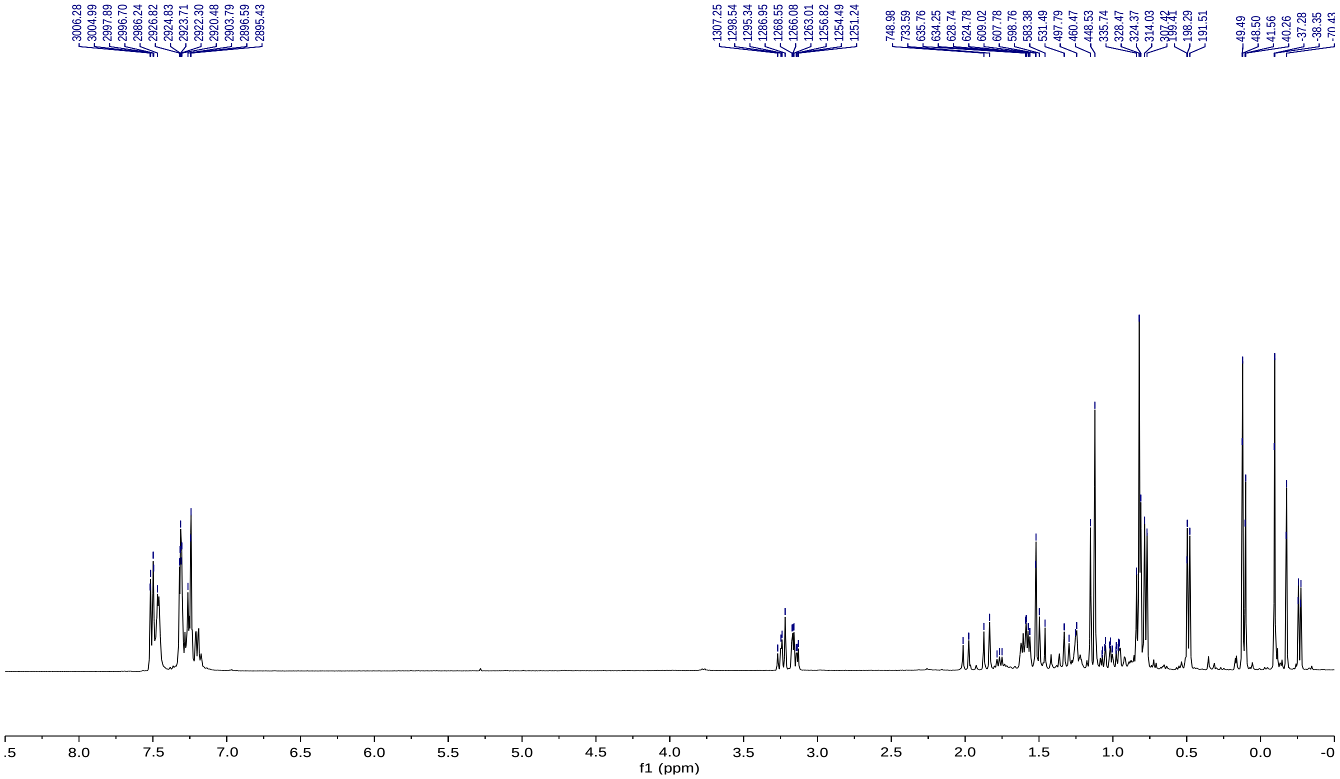

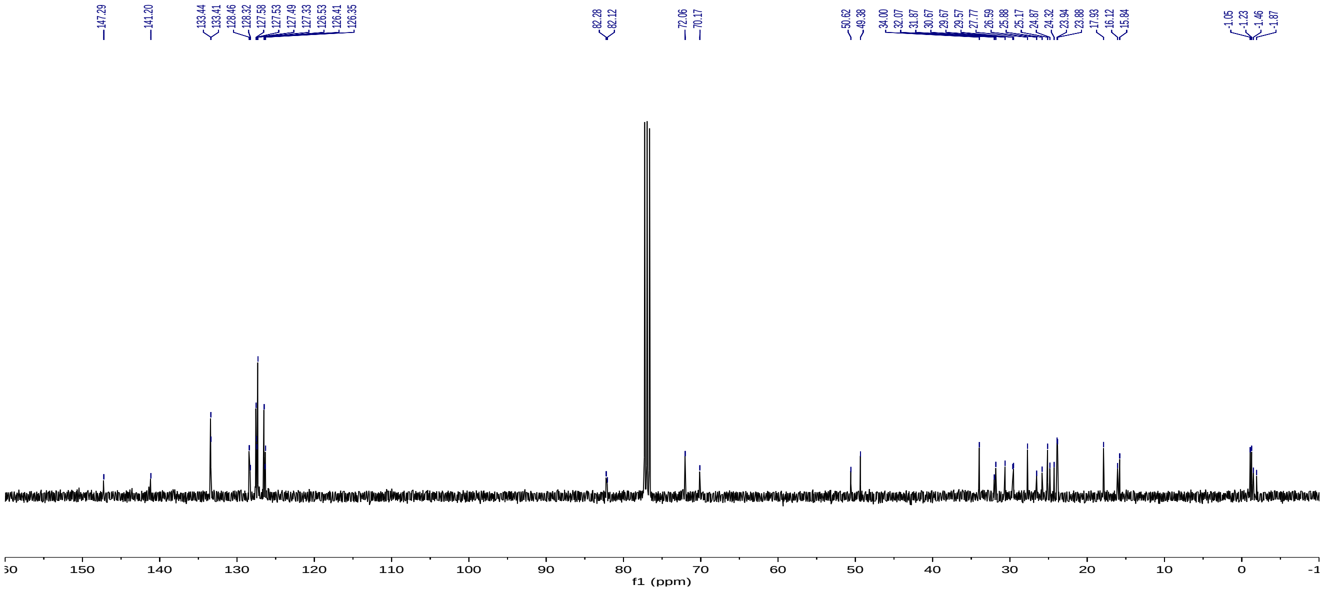

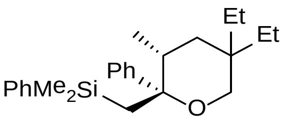

2l major (+ **3l** minor)

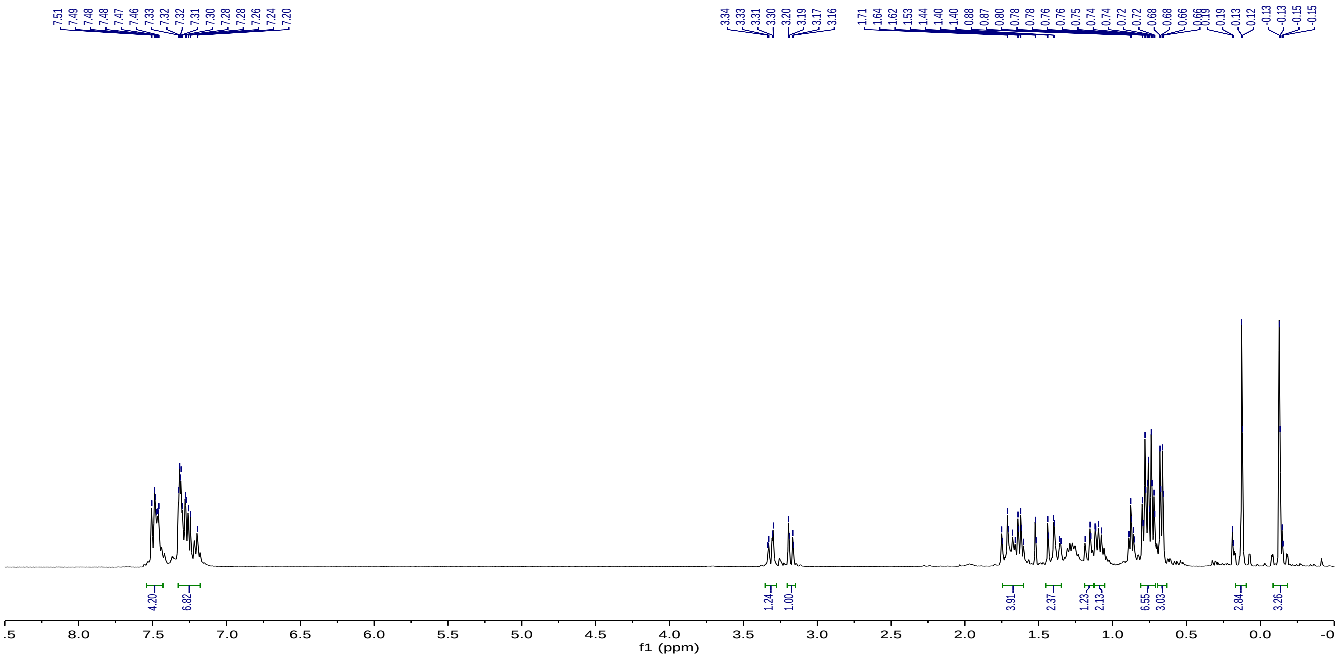

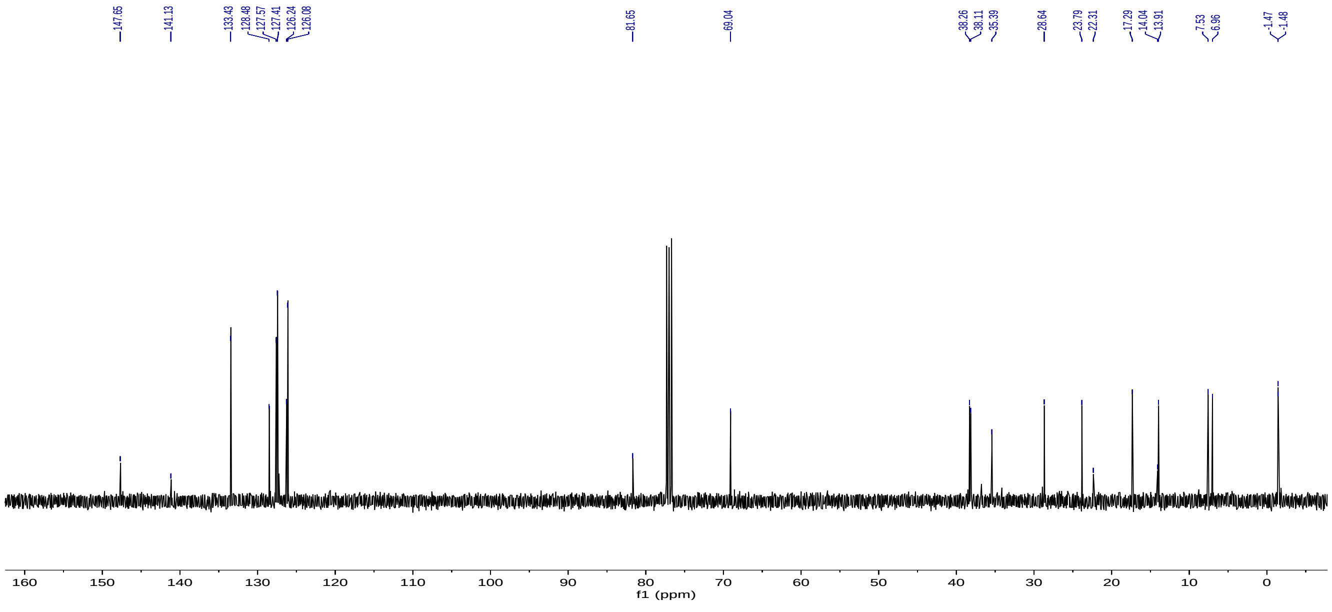

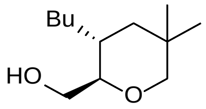
Bu
HO
O

3d

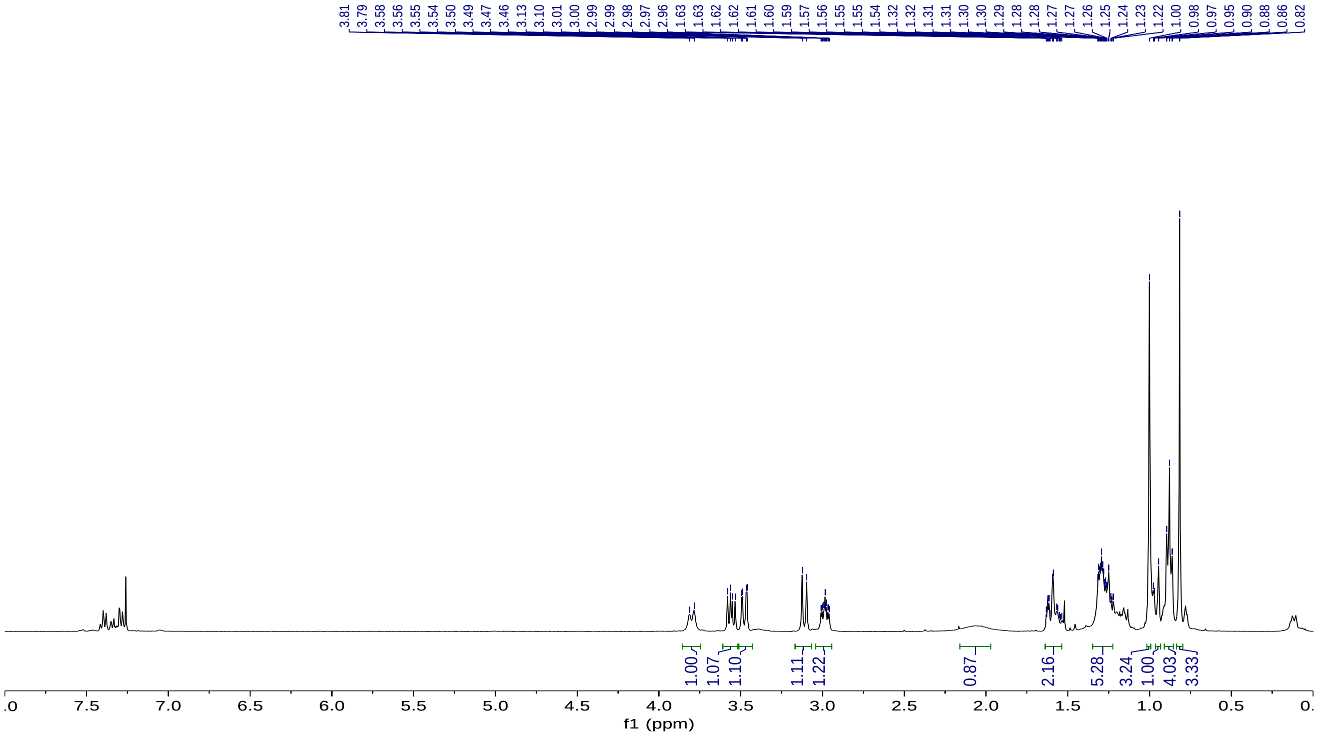
3.81 3.79 3.58 3.56 3.55 3.54 3.50 3.49 3.47 3.46 3.13 3.10 3.01 3.00 2.99 2.99 2.98 2.97 2.96 1.63 1.63 1.62 1.62 1.61 1.60 1.59 1.57 1.56 1.55 1.55 1.54 1.32 1.32 1.31 1.31 1.30 1.30 1.29 1.28 1.28 1.27 1.27 1.26 1.25 1.24 1.23 1.22 1.22 1.00 0.98 0.97 0.95 0.90 0.88 0.86 0.82
1.00 1.07 1.10 1.11 1.22 0.87 2.16 5.28 3.24 1.00 4.03 3.33
7.5 7.0 6.5 6.0 5.5 5.0 4.5 4.0 3.5 3.0 2.5 2.0 1.5 1.0 0.5
f1 (ppm)

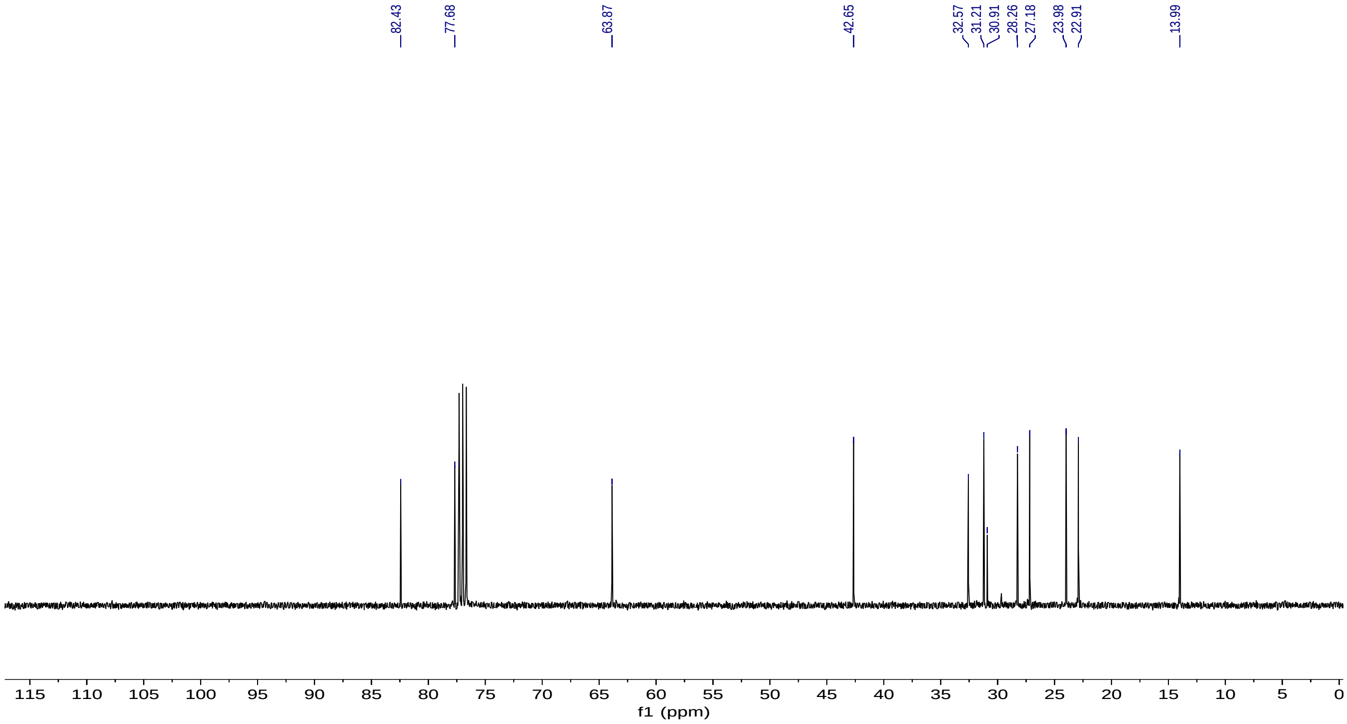
82.43 77.68 63.87 42.65 32.57 31.21 30.91 28.26 27.18 23.98 22.91 13.99
115 110 105 100 95 90 85 80 75 70 65 60 55 50 45 40 35 30 25 20 15 10 5 0
f1 (ppm)
